# Expression Analysis of Ligand-Receptor Pairs Identifies Cell-to-Cell Crosstalk between Macrophages and Tumor Cells in Lung Adenocarcinoma

**DOI:** 10.1155/2022/9589895

**Published:** 2022-09-22

**Authors:** Xiaodong Yang, Zhao An, Zhengyang Hu, Junjie Xi, Chenyang Dai, Yuming Zhu

**Affiliations:** ^1^Department of Thoracic Surgery, Shanghai Pulmonary Hospital, Tongji University, Shanghai, China; ^2^Department of Thoracic Surgery, Zhongshan Hospital, Fudan University, Shanghai, China

## Abstract

**Background:**

Lung adenocarcinoma is one of the most commonly diagnosed malignancies worldwide. Macrophage plays crucial roles in the tumor microenvironment, but its autocrine network and communications with tumor cell are still unclear.

**Methods:**

We acquired single-cell RNA sequencing (scRNA-seq) (*n* = 30) and bulk RNA sequencing (*n* = 1480) samples of lung adenocarcinoma patients from previous literatures and publicly available databases. Various cell subtypes were identified, including macrophages. Differentially expressed ligand-receptor gene pairs were obtained to explore cell-to-cell communications between macrophages and tumor cells. Furthermore, a machine-learning predictive model based on ligand-receptor interactions was built and validated.

**Results:**

A total of 159,219 single cells (18,248 tumor cells and 29,520 macrophages) were selected in this study. We identified significantly correlated autocrine ligand-receptor gene pairs in tumor cells and macrophages, respectively. Furthermore, we explored the cell-to-cell communications between macrophages and tumor cells and detected significantly correlated ligand-receptor signaling pairs. We determined that some of the hub gene pairs were associated with patient prognosis and constructed a machine-learning model based on the intercellular interaction network.

**Conclusion:**

We revealed significant cell-to-cell communications (both autocrine and paracrine network) within macrophages and tumor cells in lung adenocarcinoma. Hub genes with prognostic significance in the network were also identified.

## 1. Introduction

Lung cancer remains the leading cause of cancer incidence and death worldwide; lung adenocarcinoma is the largest subtype with increasing incidence [1–3]. Previous studies have suggested that the tumor microenvironment, including that of lung adenocarcinoma, plays crucial roles in the different steps of tumorigenesis and therapeutic responses [4–7]. The function of macrophages has been reported to be altered in lung cancer [8]. Ohtaki et al. revealed that CD204+ macrophages represented a tumor-promoting phenotype in lung adenocarcinoma [9]. Lavin et al. determined that tumor-associated macrophages had a distinct transcriptional signature in lung adenocarcinoma and summarized their immunosuppressive role in early stages of the disease [10]. However, the network of cell-to-cell communications (both autocrine and paracrine network) within macrophages and tumor cells in lung adenocarcinoma has not been fully explored. The communications among different cells are regulated by pairs of ligand and cell-surface receptor.

In recent decades, gene profiling of cancers has primarily depended on RNA sequencing (RNA-seq) technology, in which samples are regarded as a whole. Tumors, together with the tumor microenvironment, are comprised of heterogeneous cell populations, including macrophages, T cells, and cancer cells. However, bulk RNA-seq measures the averaged expression level across all cell subtypes, which fails to reflect the intrinsic heterogeneities of gene profiling and functional features [11]. Single-cell RNA sequencing (scRNA-seq) enables investigations of the tumor microenvironment at a single-cell level rather than cell-population level [12–14]. Therefore, the applications of scRNA-seq allow us to go a step further in analyzing cell-to-cell crosstalk within macrophages and tumor cells.

In this study, we explored the coexpression of ligand-receptor pairs by both RNA-seq and 10× genomics single-cell RNA sequencing (10× scRNA-seq) data, which might provide us a framework to investigate the cell-to-cell communications within macrophages and tumor cells in lung adenocarcinoma. We identified differentially expressed genes of ligand-receptor pairs in both autocrine and paracrine network within macrophages and tumor cells. Their clinical significance was also tested in lung adenocarcinoma using a machine learning model.

## 2. Methods

### 2.1. Study Cohorts

We integrated three independent cohorts of scRNA-seq data as the main study population. One was composed of tumor samples based on 14 patients with primary lung adenocarcinoma from previous literature following relevant data availability statement [15]. The other two cohorts of scRNA-seq samples were downloaded from ArraryExpress (https://www.ebi.ac.uk/arrayexpress/) database (accession numbers: E-MTAB-6149 and E-MTAB-6653) based on previous literatures. Detailed clinicopathological characteristics of all patients enrolled in the scRNA-seq cohort were showed in Supplement Table 1.

Five Gene Expression Omnibus (GEO, https://www.ncbi.nlm.nih.gov/geo/) datasets (GSE30219, GSE31210, GSE50081, GSE37745, and GSE68465) were enrolled in this study. The first four datasets were derived from the GPL570 GeneChip of Affymetrix (Santa Clara, CA, USA), while GSE68465 was based on GPL96 GeneChip of Affymetrix. Raw data and GeneChip files were downloaded directly from GEO database. For data integration of different datasets, we adopted a robust multichip average method based on RMAExpress for background adjustment, quantile normalization, and summary for gene profiling [16–18]. The GPL570 GEO cohort (544 patients) was adopted for the correlation and prognostic analyses of ligand-receptor pair genes, and the GPL96 cohort (443 patients) was used as the training cohort for the construction of the machine-learning prognostic model. Moreover, level 3 RNA-seq data of lung adenocarcinoma patients were also downloaded from The Cancer Genome Atlas (TCGA) before October 6, 2019 (https://portal.gdc.cancer.gov/). A total of 493 tumor samples were obtained with complete follow-up information. We chose the TCGA dataset as the validation cohort for the construction of machine-learning prognostic model. Detailed baseline features of all patients from both GEO and TCGA database were listed in Supplement Table 2.

### 2.2. Analyses of 10× scRNA-Seq Data

The detailed methods of 10× scRNA-seq and data preprocessing are described in the Supplement Methods. The normalized 10× scRNA-seq data was transformed into a Seurat object using the Seurat R package [19]. Principal component analysis (PCA) was performed based on the top 2000 highly variable genes. To integrate three 10× scRNA-seq cohorts in this study, we used the Harmony R package. Uniform manifold approximation and projection (UMAP) was conducted for cell clustering and visualization (Supplement Figure 1). The identifications of different cell subtypes were achieved using the CellMarker dataset and the SingleR R package [20, 21]. According to the literature, *EPCAM*, *SOX4*, and *MDK* are considered as gene markers for tumor cells, while *SFTPD*, *AGR3*, and *FOLR1* are closely associated with epithelial cells [14]. Owing to the distinct subtypes of myeloid cells, we used *CD163*, *LYZ*, *ELANE*, and *FCER1A* to differentially identify macrophages, Langerhans cells, and granulocytes [10, 20, 21]. Detailed information of the cell typing markers is shown in Supplement Figures 2 and 3.

### 2.3. Cell-to-Cell Communication Analyses

In this step, we basically followed the steps as described in the previous literatures [22, 23]. The list of ligand-receptor pairs was downloaded from the FANTOM5 project [24].

First, we explored the network of autocrine ligand-receptor gene pairs in tumor cells and macrophages. The expressions of ligand or receptor genes were compared between lung adenocarcinoma cells and normal epithelial cells using the MAST package in the scRNA-seq cohort [25]. Then, we selected pairs of ligand-receptor genes that were concurrently upregulated or downregulated in lung adenocarcinoma cells or tumor-associated macrophages. To quantify the coexpression levels of ligand-receptor pairs, Spearman's rank correlation coefficients were adopted for calculations in the bulk RNA-seq cohort (the GPL570 GEO cohort). We selected a coefficient value of 0.3 as the threshold for further screening. Gene set variation analysis (GSVA) with the Hallmark gene set was conducted to detect changes of enriched pathways [26].

Second, we explored the paracrine network of crosstalk between macrophages and lung adenocarcinoma cells. Comparisons of ligand or receptor gene expressions were also performed in macrophages stratified by its neoplastic and nonneoplastic origins in the scRNA-seq cohort. Then, we selected ligand-receptor pair genes that were separately highly expressed in these two types of cells. Subsequent correlation analyses in the bulk RNA-seq cohort and coefficient threshold were the same as above. Furthermore, we selected pathways of Hallmark and Kyoto Encyclopedia of Genes and Genomes (KEGG) which contain selected top ligand-receptor gene pairs in the above analyses. We comprehensively studied the expression changes of genes in the selected pathways in the scRNA-seq cohort. Here, we aimed to observe the transcriptional consequences at the single-cell level of ligand-receptor pathways activation. Also, the Gene Ontology (GO) analyses were performed based on selected ligand-receptor genes.

Third, we displayed the potential roles and interactions of ligand-receptor gene pairs within tumor cells and subtypes of macrophages. To calculate the M1/M2 polarization and pro-/anti-inflammatory potential of macrophages cells, we retrieved associated gene sets following previous literatures [12, 27]. In the scRNA-seq cohort, we classified and annotated subclusters of tumor-associated macrophages [15]. Based on the significantly differentially expressed ligand and receptor gene pairs in the scRNA-seq cohort, we evaluated the interaction scores of gene pairs within tumor cells and subtypes of macrophages by toolkit CellChat [15, 28].

### 2.4. Construction of the Machine-Learning Model

The Extreme Gradient Boosting (XGBoost) method is an advanced machine-learning algorithm based on the Gradient Boosting framework, which has been widely adopted.

XGBoost enhances upon the base Gradient Boosting framework by systematic and algorithmic optimizations. XGBoost provides a parallel tree boosting for effective prediction, which has been proven in many cases [29–31]. Details of the XGBoost algorithm can be obtained elsewhere (https://xgboost.readthedocs.io/en/latest/). The GEO GPL96 (GSE68465) dataset was split into low-risk (I-II stage patients) and high-risk (III-IV stage patients) groups for machine-learning predictions. Then, it was randomly divided into a training and internal test cohort with a ratio of approximately 2 : 1. We adopted significantly differentially expressed ligands or receptors in the scRNA-seq analyses as the initial gene set, and then selected those genes with prognostic values in the GSE684865 cohort. The sklearn package of Python was adopted to establish the machine-learning model based on the selected gene set. Finally, the TCGA dataset was used as the validation cohort for the machine-learning model evaluation.

### 2.5. Validations of Hub Ligand-Receptor Gene Pairs in Tumor Cells and Macrophages in Lung Adenocarcinoma

Ten lung adenocarcinoma samples and matched normal tissues were selected for validations with flow cytometry and quantitative real-time polymerase chain reaction (qRT-PCR). Experiment steps were described in previous literatures [23, 32]. Single cells of selected samples were suspended in phosphate-buffered saline with 3% fetal bovine serum and incubated with human IgG (20 *μ*g/ml, Sigma-Aldrich) for 15 minutes to remove nonspecific antibody binding. Afterwards, single cells were placed on ice and incubated with Alexa 647-conjugated mouse antihuman *EPCAM* (10 *μ*l/10^6^ cells; cat. no.: 566658, BD Biosciences, San Jose, CA, USA), PE-conjugated mouse antihuman *FOLR1* (10 *μ*l/10^6^ cells; cat. no.: FAB5646P, R&D Systems, Minneapolis, MN, USA), or Alexa 647-conjugated mouse antihuman *CD163* (10 *μ*l/10^6^ cells; cat. no.: 562669, BD Biosciences, San Jose, CA, USA) for 30 minutes. We applied Fortessa analyzer (BD Biosciences) and FACS Arial III (BD Biosciences) to quantitate and isolate stained single cells. Moreover, FlowJo software (Version 10, TreeStar, Woodburn, OR, USA) was adopted for generating and analyses. To validate the associations of selected hub ligand or receptor genes with macrophages, we adopted a public resource (Tumor IMmune Estimation Resource, TIMER) by computational approaches in the TCGA cohort. We analyzed the correlations of selected hub ligand or receptor gene expression with the level of macrophage infiltrating. Moreover, the above sorted single cells were used for subsequent RNA extraction and reverse transcription by an RNA kit (Takara, Kusatsu, Japan). We tested and compared the expressions of selected hub ligand or receptor genes in lung adenocarcinoma cells, normal epithelial cells, and macrophages (Supplement Methods).

### 2.6. Statistical Analyses

All statistical analyses were performed with IBM SPSS Statistics 22.0 (IBM, Inc., Armonk, NY, USA) and R version 3.6.1 (R Foundation for Statistical Computing, Vienna, Austria). The ligand-receptor network among cells in lung adenocarcinoma were displayed by Cytoscape version 3.7.2 (https://cytoscape.org/). Survival curves were estimated and compared following the Kaplan-Meier method and the log-rank test. Patients were divided based on the median level of gene expression. A two-tailed *P* value <0.05 was set as the threshold of statistical significance.

## 3. Results

### 3.1. Cell Typing and the Identification of Tumor Cells and Macrophages

**Figure 1:**
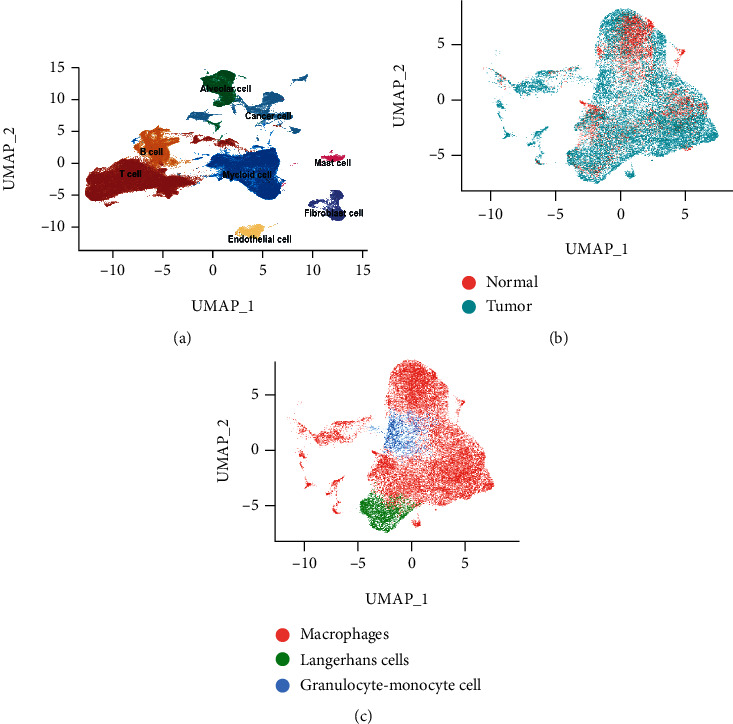
(a) Overview distribution of the 159,219 single cells stratified by the identified cell subtypes (red cluster: T cells, orange cluster: B cells, green cluster: alveolar cells, blue cluster: myeloid cells, yellow cluster: endothelial cells, pink cluster: mast cells, purple cluster: fibroblast cells, and turquoise cluster: cancer cells). (b) Overview distribution of myeloid cells stratified by the sample types (normal and tumor samples). (c) Overview distribution of macrophages (red cluster), Langerhans cells (green cluster), and granulocytes (blue cluster).

After quality filtering and merging of datasets, 159,219 cells from 21 patients (23 lung adenocarcinoma samples and 7 normal lung tissue samples) were identified based on 10× scRNA-seq (Figure 1(a) and Supplement Figure 1C). A total of 122,082 cells (76.7%) were derived from lung adenocarcinoma samples and 37,137 cells (23.3%) originated from normal lung tissue. The whole single-cell cohort was then classified into clusters using the PCA and UMAP algorithms. Subsequently, the displayed cell clusters were further distinguished by marker genes. We identified single cells in the alveolar cluster (14,712 cells, 17.6% of normal samples), lung adenocarcinoma tumor cluster (18,248 cells, 97.3% of tumor samples), and macrophage cluster (29,520 cells in total, 20,379 cells of neoplastic origin and 9141 cells of nonneoplastic origin) (Figures 1(a)–1(c)).

### 3.2. Expression Correlation Analyses Suggested Significant Autocrine Ligand-Receptor Gene Pairs of Tumor Cells in Lung Adenocarcinoma

**Figure 2:**
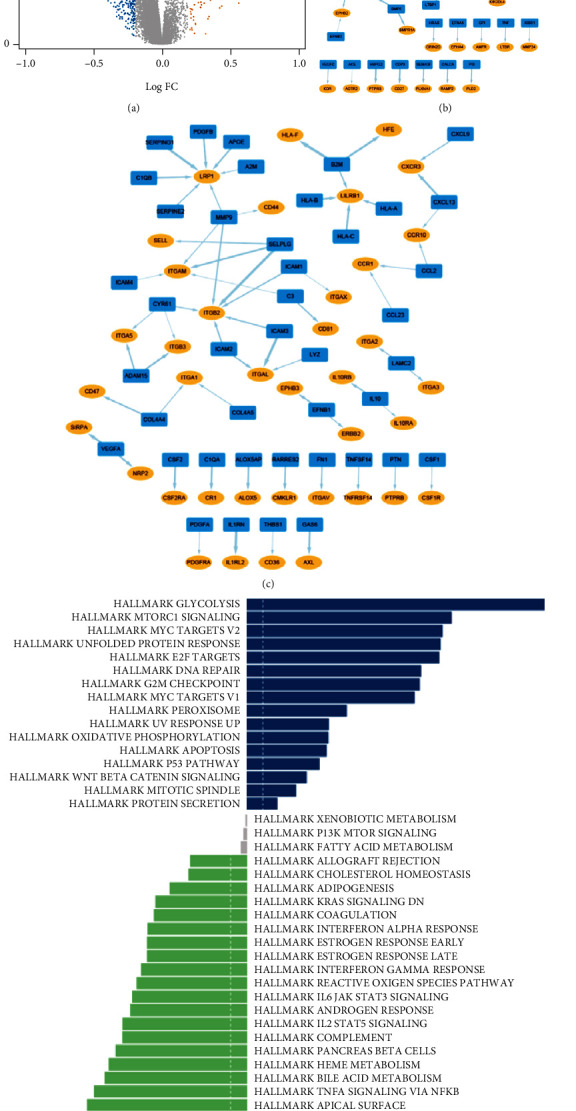
The ligand-receptor gene pairs identified in the autocrine network of lung adenocarcinoma tumor cells. (a) Volcano plot of the differentially expressed genes between tumor cells and normal epithelial cells. (b) The network of selected upregulated ligand-receptor gene pairs in tumor cells of lung adenocarcinoma (blue round rectangles: ligands; yellow ellipse shapes: receptors; line width is consistent with the correlation coefficient between the ligand and receptor). (c) The network of selected downregulated ligand-receptor gene pairs in tumor cells of lung adenocarcinoma (blue round rectangles: ligands; yellow ellipse shapes: receptors; line width: consistent with the correlation coefficient). (d) GSVA analysis of the Hallmark gene sets identifies significant functional pathways in lung adenocarcinoma cells (tumor cells vs. normal epithelial cells).

We detected 13,560 differentially expressed genes by comparing lung adenocarcinoma tumor cells and normal epithelial cells based on 10× scRNA-seq cohort (Figure 2(a)). As a result, we identified 240 upregulated and 234 downregulated ligand-receptor pair genes that were significantly increased or decreased simultaneously in lung adenocarcinoma tumor cells, which constituted the autocrine network of tumor cells. Correlation analyses were performed for each pair in the GEO GPL570 dataset. We chose 44 upregulated and 63 downregulated pairs with coefficients >0.3 (Figures 2(b) and 2(c), Supplement Table 3). The top five upregulated and downregulated gene pairs were as follows: *TGFB1*-*ENG*, *TGM2*-*TBXA2R*, *HSPG2*-*PTPRS*, *BMP5*-*ACVR2A*, and *HLA-G*-*KIR2DL4*; *B2M*-*HLA-F*, *SELPLG*-*ITGB2*, *IL1RN*-*IL1RL2*, *ICAM3*-*ITGAL*, and *SERPING1*-*LRP*1, respectively. A direct comparison of enriched pathways was conducted between tumor cells and epithelial cells in the scRNA-seq cohort. As shown in Figure 2(d), pathways associated with glycolysis, mTORC1 signaling, Myc targets, DNA repair, and G2M checkpoint were significantly enriched in tumor cells.

### 3.3. Expression Correlation Analyses Revealed Important Autocrine Ligand-Receptor Gene Pairs of Tumor-Associated Macrophages in Lung Adenocarcinoma

**Figure 3:**
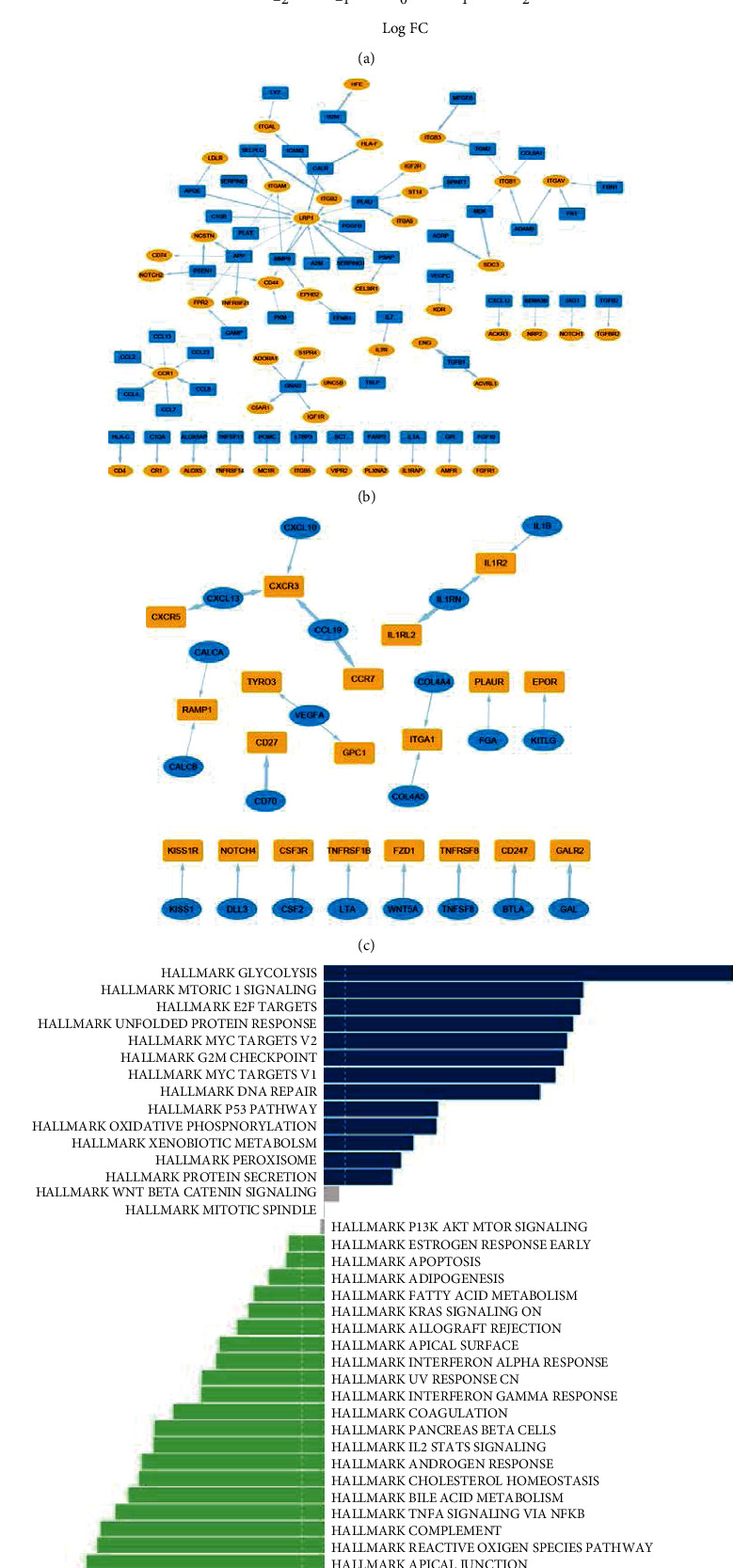
The ligand-receptor gene pairs identified in the autocrine network of macrophages. (a) Volcano plot of the differentially expressed genes between tumor-associated macrophages and nontumor-associated macrophages. (b) The network of selected upregulated ligand-receptor gene pairs in macrophages of lung adenocarcinoma (blue round rectangles: ligands; yellow ellipse shapes: receptors; line width is consistent with the correlation coefficient between the ligand and receptor). (c) The network of selected downregulated ligand-receptor gene pairs in macrophages of lung adenocarcinoma (blue round rectangles: ligands; yellow ellipse shapes: receptors; line width is consistent with the correlation coefficient between the ligand and receptor). (d) GSVA analysis of the Hallmark pathways in macrophages (tumor-associated macrophages vs. nontumor-associated macrophages).

A total of 11,192 differentially expressed genes were identified in macrophages stratified by origin (neoplastic cells vs. nonneoplastic cells) based on the 10× scRNA-seq cohort (Figure 3(a)). Similarly, 307 upregulated and 73 downregulated ligand-receptor pair genes in tumor-associated macrophages were identified, which constituted the autocrine network of tumor-associated macrophages. Correlation analyses were performed for each pair in the GEO GPL570 dataset. We detected 84 upregulated and 25 downregulated ligand-receptor pair genes with coefficients >0.3 (Figures 3(b) and 3(c), Supplement Table 3). The top five upregulated and downregulated gene pairs were as follows: *TGFB1*-*ENG*, *B2M*-*HLA-F*, *SELPLG*-*ITGB2*, *SERPING1*-*LRP1*, and *AGRP*-*SDC3*; *2PRS19*-*CCR7*, *IL1RN*-*IL1RL2*, *CCL19*-*CXCR3*, *CD70*-*CD27*, and *CXCL13*-*CXCR5*, respectively. We also compared the enriched pathways between macrophages with different origins in the scRNA-seq cohort. Glycolysis pathway was still the leading enriched pathway in tumor-associated macrophages.

### 3.4. Crosstalk between Tumor Cells and Macrophages Is Associated with Prognosis of Lung Adenocarcinoma Patients

**Figure 4:**
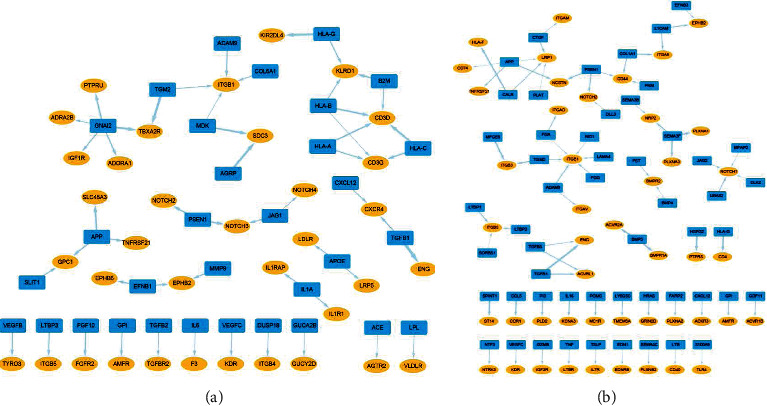
The crosstalk between macrophages and lung adenocarcinoma cells. (a) The network of selected upregulated ligand-receptor pairs from macrophages to tumor cells (blue round rectangles: ligands; yellow ellipse shapes: receptors; line width is consistent with the correlation coefficient between the ligand and receptor). (b) The network of selected upregulated ligand-receptor pairs from tumor cells to macrophages (blue round rectangles: ligands; yellow ellipse shapes: receptors; line width is consistent with the correlation coefficient between the ligand and receptor).

To assess how macrophages connect with tumor cells in lung adenocarcinoma, we chose 52 gene pairs, of which ligands in tumor-associated macrophages and receptors in tumor cells were highly expressed, respectively (Figure 4(a)). The top five upregulated gene pairs were: *TGFB1*-*ENG*, *TGM2*-*TBXA2R*, *AGRP*-*SDC3*, *HLA-G*-*KIR2DL4*, and *GNAI2*-*TBXA2R*. In total, there were 54 ligands or receptors in the network that showed prognostic significance in the GPL570 GEO cohort (Supplement Table 3). We selected pathways containing top five upregulated ligand-receptor gene pairs and analyzed the gene expression changes of these pathways in the scRNA-seq cohort. We found that there was a trend of overexpression of genes in the *TGF-β* signaling pathway of cancer cells, which suggested potential pathway activation of *TGFB1*-*ENG* at the single-cell level (Supplement Figure 4A). Then, gene functional enrichment analysis of GO suggested that the crosstalk between macrophages and tumor cells were significantly associated with cytokine productions and secretions (Supplement Figure 5A).

To evaluate how lung adenocarcinoma tumor cells communicate with macrophages, we selected 70 gene pairs, of which ligands in tumor cells and receptors in tumor-associated macrophages were upregulated, respectively (Figure 4(b)). The top five upregulated gene pairs were: *TGFB1*-*ENG*, *HSPG2*-*PTPRS*, *HLA-G*-*CD4*, *BMP5*-*ACVR2A*, and *MFGE8*-*ACVR2A*. In total, there were 80 ligands or receptors in the network that showed prognostic significance in the GPL570 GEO cohort (Supplement Table 3). We selected pathways containing top five upregulated ligand-receptor gene pairs and analyzed the gene expression changes of these pathways in the scRNA-seq cohort. We found that there was a trend of overexpression of genes in the allograft rejection, antigen processing, and presentation signaling pathways of tumor-associated macrophages, which suggested potential pathway activations of *HLA-G-CD4* at the single-cell level (Supplement Figures 4B and 4C). Then, gene functional enrichment analysis of GO indicated that the communications between tumor cells and macrophages were significantly related to lymphocytes adhesions, migrations, and differentiations (Supplement Figure 5B).

### 3.5. Heterogeneities of Interaction Roles of Ligand-Receptor Gene Pairs within Tumor Cells and Subtypes of Tumor-Associated Macrophages

**Figure 5:**
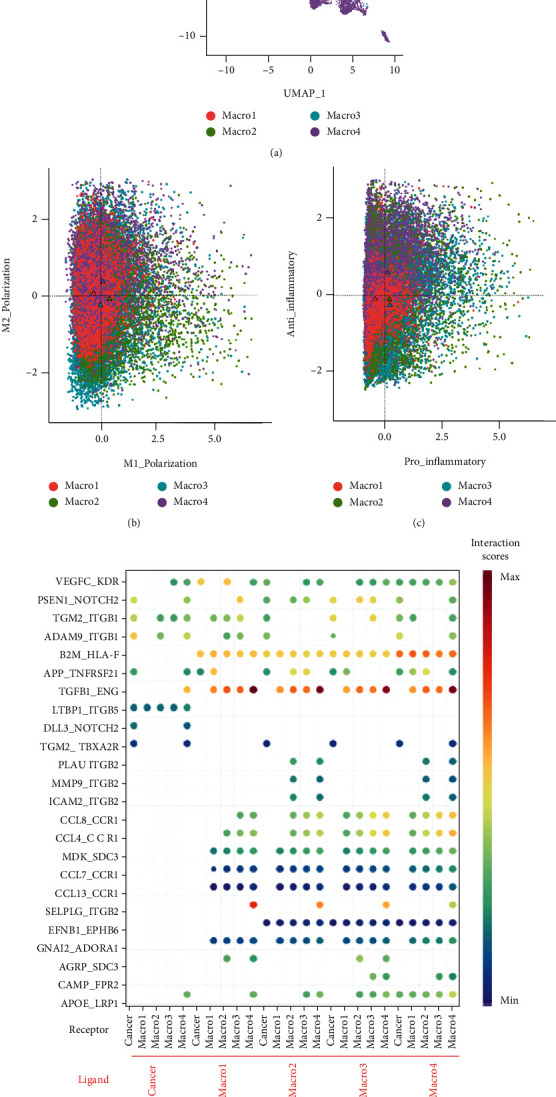
(a) UMAP plot of tumor-associated macrophages (4 subtypes identified). (b) Scatter plot showing M1 and M2 polarization scores for each color-coded subtypes of tumor-associated macrophages. (c) Scatter plot showing pro- and anti-inflammatory scores for each color-coded subtypes of tumor-associated macrophages. (d) Bubble plot showing selected ligand-receptor interactions between tumor cells and subtypes of tumor-associated macrophages. *P* value is indicated by circle size, with the scale to the right.

Considering the heterogeneities of tumor-associated macrophages, we tried to display the differences of interaction roles of ligand-receptor gene pairs in the autocrine and paracrine network of tumor cells and macrophages. In the scRNA-seq cohort, we reclustered tumor-associated macrophages and 4 subtypes were revealed in our study (Figure 5(a)). We also calculated the M1/M2 polarization and pro-/anti-inflammatory scores based on previous study [27]. The M1/M2 polarization and pro-/anti-inflammatory scores for each subtypes of macrophages were shown in Figures 5(b) and 5(c). Then, interaction scores were evaluated for the significantly differentially expressed ligand-receptor gene pairs (Figure 5(d)).

### 3.6. Machine-Learning Prognostic Model Based on Ligand-Receptor Interactions

**Figure 6:**
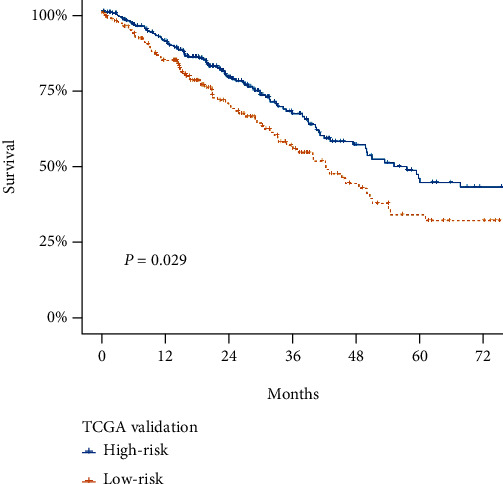
Survival analysis of TCGA lung adenocarcinoma patients stratified by machine-learning predicted high- and low-risk groups. (*P* = 0.029).

To further investigate the prognostic significance of the above ligand-receptor gene pairs, we built a machine-learning model using XGBoost algorithm. Differentially expressed ligands or receptors in the scRNA-seq analyses (Supplement Table 3) with prognostic value in the GSE68465 cohort were included for calculation. We enrolled a gene set composed of 155 genes for subsequent model construction. The entire GSE68465 cohort was randomly divided into a training and test dataset with a ratio of approximately 2 : 1. We found that the machine-learning high- and low-risk predictive model achieved a precision value of 0.94 and a recall value of 0.78 in the randomly selected test dataset based on GSE68465 cohort. We then adopted the TCGA cohort to validate the XGBoost predictive model. There was a significant prognostic difference between the predicted high- and low-risk groups in the TCGA cohort (*P* = 0.029, Figure 6).

### 3.7. Validations of Hub Ligand-Receptor Gene Pairs in Tumor Cells and Macrophages in Lung Adenocarcinoma

We used flow cytometry to validate lung adenocarcinoma cells, normal epithelial cells, and macrophages with cell markers (*EPCAM*, *FOLR1*, and *CD163*). Supplement Figure 6 shows the reliability of selected cell markers in this study. *EPCAM*+/*FOLR1*- cells had a larger proportion in tumor samples, while there were more *EPCAM*-/*FOLR1*+ cells in normal lung tissues (Supplement Figure 6A–6D). Moreover, *CD163*+ cells accounted for a larger proportion in tumor samples than normal lung tissues, which was mainly consistent with our initial results of cell typing for macrophages by scRNA-seq (Supplement Figure 6E–6H). In the TIMER database, we selected top ligand or receptor genes which were associated with the cell-to-cell paracrine or autocrine communications of macrophages. As shown in Supplement Figure 7, we found that the selected ligand or receptor genes were significantly associated with the level of macrophage infiltrating in the TCGA cohort. It proved the potential significance of ligand and receptor genes in macrophages based on previous scRNA-seq investigations. Furthermore, we investigated the expression level of selected ligand or receptor genes in the above sorted cells by qRT-PCR. We found that *TGFB1*, *ENG*, *TGM2*, *TBXA2R*, *HSPG2*, and *PTPRS* were significantly upregulated in lung adenocarcinoma cells (Supplement Figure 8A). Also, *TGFB1*, *ENG*, *B2M*, *HLA-F*, *SELPLG*, and *ITGB2* were significantly increased in tumor-associated macrophages, compared with those nontumor-associated macrophages (Supplement Figure 8B). These findings were similar to the scRNA-seq results which effectively explored the differentially expressed genes. And the identified ligand or receptor genes in scRNA-seq analyses showed significant expression changes in tumor cells and tumor-associated macrophages.

## 4. Discussion

An increasing number of studies have revealed the crucial roles of the tumor microenvironment in cancer proliferation, invasion, metastasis, and therapeutic efficacy, especially in lung adenocarcinoma [6, 33, 34]. However, the most commonly used bulk RNA-seq fails to reflect intrinsic expression differences and cell heterogeneities within tumors and in the surrounding stromal cells. Moreover, cell-to-cell crosstalk within the tumor microenvironment has not been fully investigated. The establishment of multicellular gene network may facilitate to identify promising biomarkers for predicting prognosis and therapeutic resistance of cancer patients [35, 36]. We are now able to explore cell-to-cell communications of lung adenocarcinoma as a result of scRNA-seq [14, 34]. Here, we explored the network of cell-to-cell crosstalk within lung adenocarcinoma cells and macrophages based on analyzing coexpressions of ligand-receptor pairs.

The tumor microenvironment is of great importance in promoting tumor proliferation, invasion, and metastasis. Macrophages are enriched in the core site and play roles in biological functions, such as migration, metabolism, and polarization [37]. First, we explored the network of autocrine ligand-receptor gene pairs of tumor cells in lung adenocarcinoma. We found that *TGFB1* and its binding partner *ENG* were both highly expressed in tumor cells and *TGFB1*-*ENG* gene pair occupied a key position in the network (Figure 2(b)). The comparison of enriched pathways between tumor cells and normal epithelial cells were consistent with previous literatures with respect to cancer proliferation, invasion, and metastasis [38–41]. Second, we analyzed the network of autocrine ligand-receptor gene pairs of macrophages in lung adenocarcinoma. Some of the selected ligand or receptor genes were found to have potential vital roles in the communications, which were similar to previous literatures. For example, *LRP1* is an endocytic and cell-signaling receptor that regulates cell migration. Staudt et al. observed that *LRP1* mediated macrophage recruitment and angiogenesis in tumors [42]. In this study, we detected the significant role of *LRP1* in the network of macrophage autocrine signaling (Figure 3(b)). Also, previous research has shown that *CXCR3* is correlated with decreased M2 macrophage infiltration and a favorable prognosis in gastric cancer. Our results indicated that *CXCR3* was downregulated in tumor-associated macrophages (Figure 3(c)) [43]. In the differentiated pathway analyses, glycolysis pathway was the leading one in tumor-associated macrophages. Studies have found the significant roles of immunometabolism in the tumor microenvironment, suggesting potential therapeutic implications [44–47]. Finally, we established the network of crosstalk between tumor cells and macrophages in lung adenocarcinoma. In this study, we found that overexpression of *TGM2* conferred a significantly worse survival in the GPL560 GEO cohort and had an active part in the paracrine crosstalk (Figure 4(a) and Supplement Table 3). Furthermore, we identified M1/M2 polarization and pro-/anti-inflammatory tumor-associated macrophages based on previous studies [27]. Then, we explored potential associations of ligand-receptor gene pairs with cell-to-cell communications among subclusters of cells (tumor cell and macrophage). In addition, we established a machine-learning model to predict survival based on identified ligand-receptor pairs in lung adenocarcinoma. Good performance in both test and validation cohorts suggested the significance of autocrine and paracrine in tumorigenesis and progression. Taken together, our study provides a landscape of the autocrine interactions and cell-to-cell communications within macrophages and tumor cells, which may help guide future experiments.

Traditional bulk RNA-seq fails to reveal the heterogeneity of gene profiling and tumor-infiltrating cells [11]. Recently, silico algorithms have been developed to estimate the tumor microenvironment using bulk RNA-seq; however, these methods are still not as direct and thorough as scRNA-seq [48, 49]. The use of scRNA-seq may provide new insight about new potential targets or cell-specific abnormally expressed genes. For example, we observed that *PLXNA1*, *PLXNA2*, and *PLXNA3* were all significantly associated with prognosis in the GPL570 GEO dataset (Supplement Table 3). However, few studies have focused on the plexin-A family in terms of cancer progression or tumor-associated macrophages. The advancements of scRNA-seq have greatly facilitated novel approaches for precision and translational medicine [50]. For example, Kim et al. adopted scRNA-seq and extensively showed the molecular and cellular dynamics in metastatic lung adenocarcinoma [51]. Kim et al. detected the transformation of proinflammatory monocytes into macrophages with cells losing their proinflammatory nature and gaining anti-inflammatory signatures by trajectory analyses [51]. The identifications of transitions and subpopulations during the process revealed potential targets in cancer-microenvironment interactions.

There were limitations of this research that should be mentioned. We investigated the crosstalk of autocrine and paracrine networks of macrophages and validated the strategies we employed in the scRNA-seq analyses by flow cytometry and qRT-PCR. However, the detailed mechanisms of these ligands and receptors will require further validations *in vitro* and *in vivo*, such as immunofluorescence. Moreover, the activations of ligand-receptor or downstream pathways require confirmations to the communications between macrophages and tumor cells. Thorough experimental plans may be needed, especially for top listed ligand-receptor pairs. The above validations could further lead to identity effective signatures with predictive value for survival and therapeutic resistance [35]. Furthermore, with the extensive applications of scRNA-seq, increasing tools were built to model not only functional intercellular communications but also intracellular gene regulatory network, such as scMLnet [52]. These tools may facilitate us in the establishment of crosstalk network. More importantly, the extensive morphologic heterogeneities among tumors, including tumor cellularity, extent, and compositions of matrix and vascularity, should also be further considered, which requires highly precise evaluation and extraction process of single cells. Kim et al. collected different samples, like pleural fluids and lymph node or brain metastases, to elucidate the cellular dynamics in LUAD progression [51]. However, we still need to consider the differences of clinicopathological features among patients, like EGFR mutation status and ground-glass imaging feature, which will require a larger study population.

## 5. Conclusion

We explored the landscape of cell-to-cell communication and crosstalk between macrophages and tumor cells in lung adenocarcinoma. Hub genes with prognostic significance in the network were also identified. The machine-learning predictive model showed the significance of ligand and receptor genes in tumor progression.

## Data Availability

The datasets generated and/or analyzed during the current study are available from previous literatures listed in the references, public datasets, and the corresponding authors on reasonable request.

## Supplementary Materials

Supplementary Materials Supplement Figure 1: A. The integration of single-cell data with Harmony shows the sample corresponding cohort (red cluster: samples from E-MTAB-6149; green cluster: samples from E-MTAB-6653; blue cluster: samples from previous literatures). B. Three scRNA-seq are well integrated in the first 2 dimensions after Harmony. C. Overview distribution of the 159,219 single cells from 18 lung adenocarcinoma samples and 7 normal tissue samples (red cluster: normal samples; turquoise cluster: tumor samples). Supplement Figure 2: Expression of the cell typing marker genes for identifying tumor cells, alveolar cells, and macrophages. Supplement Figure 3: A. Dot plot of the expression of marker genes for cell subtypes. B. Dot plot of the expression of marker genes for macrophages. Supplement Figure 4. A. Heatmap of gene expression in the Hallmark *TGF-β* signaling pathway stratified by cell types in the scRNA-seq. B. Heatmap of gene expression in the KEGG allograft rejection signaling pathway stratified by cell types in the scRNA-seq. C. Heatmap of gene expression in the KEGG antigen processing and presentation signaling pathway stratified by cell types in the scRNA-seq. Supplement Figure 5. A. GO analysis for selected ligand-receptor genes in the crosstalk from macrophages to lung adenocarcinoma cells. B. GO analysis for selected ligand-receptor genes in the crosstalk from lung adenocarcinoma cells to macrophages. Supplement Figure 6: Identified and sorted the key cell marker genes in normal epithelial cells, lung adenocarcinoma cells, and macrophages by flow cytometry. A, B. *FOLR1*+/*EPCAM*- cells accounted for larger proportions than *FOLR1*-/*EPCAM*+ in normal lung samples (0.30% vs 1.95%, 0.19 vs 1.32%) (*X*-axis: PE-conjugated mouse antihuman *FOLR1*, *Y*-axis: Alexa 647-conjugated mouse antihuman *EPCAM*). C, D. *FOLR1*-/*EPCAM*+ cells accounted for larger proportions than *FOLR1*+/*EPCAM*- in lung adenocarcinoma samples (10.4% vs 2.03%, 17.1 vs 1.47%) (*X*-axis: PE-conjugated mouse antihuman *FOLR1*, *Y*-axis: Alexa 647-conjugated mouse antihuman *EPCAM*). E, F. *CD163*+ cells (macrophages) accounted for 2.66% and 3.86% in normal lung samples (*X*-axis: Alexa 647-conjugated mouse antihuman *CD163*). G, H. *CD163*+ cells (macrophages) accounted for 6.86% and 6.65% in lung adenocarcinoma samples (*X*-axis: Alexa 647-conjugated mouse anti-human *CD163*). Supplement Figure 7: Associations of the expressions of selected ligand or receptor genes with macrophage infiltrating in lung adenocarcinoma of the TCGA cohort by TIMER database. A. *TGFB1* (*P* < 0.05, Spearman's *ρ* = 0.261). B. *ENG* (*P* < 0.05, Spearman's *ρ* = 0.293). C. *B2M* (*P* < 0.05, Spearman's *ρ* = 0.175). D. *HLA-F* (*P* > 0.05, Spearman's *ρ* = −0.082). E. *SELPLG* (*P* < 0.05, Spearman's *ρ* = 0.321). F. *ITGB2* (*P* < 0.05, Spearman's *ρ* = 0.293). G. *TGM2* (*P* < 0.05, Spearman's *ρ* = 0.273). H. *AGRP* (*P* < 0.05, Spearman's *ρ* = 0.163). I. *PTPRS* (*P* < 0.05, Spearman's *ρ* = 0.169). J. *CD4* (*P* < 0.05, Spearman's *ρ* = 0.43). Supplement Figure 8: Validations of the expression changes of top ligand or receptor genes in lung adenocarcinoma cells and tumor-associated macrophages. A. The mRNA relative expressions level of *TGFB1*, *ENG*, *TGM2*, *TBXA2R*, *HSPG2*, and *PTPRS* were significantly increased in lung adenocarcinoma cells than normal epithelial cells. B. The mRNA relative expressions of *TGFB1*, *ENG*, *B2M*, *HLA-F*, *SELPLG*, and *ITGB2* were significantly increased in tumor-associated macrophages than macrophages. Supplement Method: The detailed methods of 10× scRNA-seq and data preprocessing. Supplement Table 1: Characteristics of the 21 LUAD patients included in this study for scRNA-seq analysis. Supplement Table 2: Baseline characteristics of enrolled patient cohorts from GEO and TCGA databases. Supplement Table 3: Identified significant ligand-receptor gene pairs in the cell-to-cell communications within tumor cells and macrophages in lung adenocarcinoma.

## References

[1] Bray F., Ferlay J., Soerjomataram I., Siegel R. L., Torre L. A., Jemal A. (2018). Global cancer statistics 2018: GLOBOCAN estimates of incidence and mortality worldwide for 36 cancers in 185 countries. *CA: a Cancer Journal for Clinicians*.

[2] Siegel R. L., Miller K. D., Jemal A. (2019). Cancer statistics, 2019. *CA: a Cancer Journal for Clinicians*.

[3] Lu T., Yang X., Huang Y. (2019). Trends in the incidence, treatment, and survival of patients with lung cancer in the last four decades. *Cancer Management and Research*.

[4] Chen Y. P., Zhang Y., Lv J. W. (2017). Genomic analysis of tumor microenvironment immune types across 14 solid cancer types: immunotherapeutic implications. *Theranostics*.

[5] Jing X., Yang F., Shao C. (2019). Role of hypoxia in cancer therapy by regulating the tumor microenvironment. *Molecular Cancer*.

[6] Yang X., Shi Y., Li M. (2019). Identification and validation of an immune cell infiltrating score predicting survival in patients with lung adenocarcinoma. *Journal of Translational Medicine*.

[7] Pitt J. M., Marabelle A., Eggermont A., Soria J. C., Kroemer G., Zitvogel L. (2016). Targeting the tumor microenvironment: removing obstruction to anticancer immune responses and immunotherapy. *Annals of Oncology*.

[8] Pouniotis D. S., Plebanski M., Apostolopoulos V., McDonald C. F. (2006). Alveolar macrophage function is altered in patients with lung cancer. *Clinical and Experimental Immunology*.

[9] Ohtaki Y., Ishii G., Nagai K. (2010). Stromal macrophage expressing CD204 is associated with tumor aggressiveness in lung adenocarcinoma. *Journal of Thoracic Oncology*.

[10] Lavin Y., Kobayashi S., Leader A. (2017). Innate immune landscape in early lung adenocarcinoma by paired single-cell analyses. *Cell*.

[11] Kulkarni A., Anderson A. G., Merullo D. P., Konopka G. (2019). Beyond bulk: a review of single cell transcriptomics methodologies and applications. *Current Opinion in Biotechnology*.

[12] Azizi E., Carr A. J., Plitas G. (2018). Single-cell map of diverse immune phenotypes in the breast tumor microenvironment. *Cell*.

[13] Aoki T., Chong L. C., Takata K. (2020). Single cell transcriptome analysis reveals disease-defining T cell subsets in the tumor microenvironment of classic Hodgkin lymphoma. *Cancer Discovery*.

[14] Lambrechts D., Wauters E., Boeckx B. (2018). Phenotype molding of stromal cells in the lung tumor microenvironment. *Nature Medicine*.

[15] Chen Z., Huang Y., Hu Z. (2021). Landscape and dynamics of single tumor and immune cells in early and advanced-stage lung adenocarcinoma. *Clinical and Translational Medicine*.

[16] Bolstad B. M., Irizarry R. A., Astrand M., Speed T. P. (2003). A comparison of normalization methods for high density oligonucleotide array data based on variance and bias. *Bioinformatics*.

[17] Irizarry R. A., Bolstad B. M., Collin F., Cope L. M., Hobbs B., Speed T. P. (2003). Summaries of Affymetrix GeneChip probe level data. *Nucleic Acids Research*.

[18] Irizarry R. A., Hobbs B., Collin F. (2003). Exploration, normalization, and summaries of high density oligonucleotide array probe level data. *Biostatistics*.

[19] Macosko E. Z., Basu A., Satija R. (2015). Highly parallel genome-wide expression profiling of individual cells using Nanoliter droplets. *Cell*.

[20] Zhang X., Lan Y., Xu J. (2019). CellMarker: a manually curated resource of cell markers in human and mouse. *Nucleic Acids Research*.

[21] Aran D., Looney A. P., Liu L. (2019). Reference-based analysis of lung single-cell sequencing reveals a transitional profibrotic macrophage. *Nature Immunology*.

[22] Yuan D., Tao Y., Chen G., Shi T. (2019). Systematic expression analysis of ligand-receptor pairs reveals important cell-to-cell interactions inside glioma. *Cell Communication and Signaling: CCS*.

[23] Chen Z., Yang X., Bi G. (2020). Ligand-receptor interaction atlas within and between tumor cells and T cells in lung adenocarcinoma. *International Journal of Biological Sciences*.

[24] Ramilowski J. A., Goldberg T., Harshbarger J. (2015). A draft network of ligand-receptor-mediated multicellular signalling in human. *Nature Communications*.

[25] Finak G., McDavid A., Yajima M. (2015). MAST: a flexible statistical framework for assessing transcriptional changes and characterizing heterogeneity in single-cell RNA sequencing data. *Genome Biology*.

[26] Hanzelmann S., Castelo R., Guinney J. (2013). GSVA: gene set variation analysis for microarray and RNA-seq data. *BMC Bioinformatics*.

[27] Sun Y., Wu L., Zhong Y. (2021). Single-cell landscape of the ecosystem in early-relapse hepatocellular carcinoma. *Cell*.

[28] Jin S., Guerrero-Juarez C. F., Zhang L. (2021). Inference and analysis of cell-cell communication using CellChat. *Nature Communications*.

[29] Xie Y., Zhang C., Hu X. (2020). Machine learning assisted synthesis of metal-organic Nanocapsules. *Journal of the American Chemical Society*.

[30] Kilic A., Goyal A., Miller J. K. (2020). Predictive utility of a machine learning algorithm in estimating mortality risk in cardiac surgery. *The Annals of Thoracic Surgery*.

[31] Polano M., Chierici M., Dal Bo M. (2019). A pan-cancer approach to predict responsiveness to immune checkpoint inhibitors by machine learning. *Cancers*.

[32] Chen Z., Zhao M., Li M. (2020). Identification of differentially expressed genes in lung adenocarcinoma cells using single-cell RNA sequencing not detected using traditional RNA sequencing and microarray. *Laboratory Investigation*.

[33] Zeng D., Li M., Zhou R. (2019). Tumor microenvironment characterization in gastric cancer identifies prognostic and immunotherapeutically relevant gene signatures. *Cancer Immunology Research*.

[34] Jiang Y., Zhang Q., Hu Y. (2018). ImmunoScore signature: a prognostic and predictive tool in gastric cancer. *Annals of Surgery*.

[35] Zhang J., Guan M., Wang Q., Zhang J., Zhou T., Sun X. (2020). Single-cell transcriptome-based multilayer network biomarker for predicting prognosis and therapeutic response of gliomas. *Briefings in Bioinformatics*.

[36] Sun X., Liu X., Xia M., Shao Y., Zhang X. D. (2019). Multicellular gene network analysis identifies a macrophage-related gene signature predictive of therapeutic response and prognosis of gliomas. *Journal of Translational Medicine*.

[37] Dehne N., Mora J., Namgaladze D., Weigert A., Brune B. (2017). Cancer cell and macrophage cross-talk in the tumor microenvironment. *Current Opinion in Pharmacology*.

[38] Cantelmo A. R., Conradi L. C., Brajic A. (2016). Inhibition of the glycolytic activator PFKFB3 in endothelium induces tumor vessel normalization, impairs metastasis, and improves chemotherapy. *Cancer Cell*.

[39] Banerjee D., Gorlick R., Liefshitz A. (2000). Levels of E2F-1 expression are higher in lung metastasis of colon cancer as compared with hepatic metastasis and correlate with levels of thymidylate synthase. *Cancer Research*.

[40] Macerelli M., Ganzinelli M., Gouedard C. (2016). Can the response to a platinum-based therapy be predicted by the DNA repair status in non-small cell lung cancer?. *Cancer Treatment Reviews*.

[41] Gordan J. D., Thompson C. B., Simon M. C. (2007). HIF and c-Myc: sibling rivals for control of cancer cell metabolism and proliferation. *Cancer Cell*.

[42] Staudt N. D., Jo M., Hu J. (2013). Myeloid cell receptor LRP1/CD91 regulates monocyte recruitment and angiogenesis in tumors. *Cancer Research*.

[43] Chen F., Yuan J., Yan H., Liu H., Yin S. (2019). Chemokine receptor CXCR3 correlates with decreased M2 macrophage infiltration and favorable prognosis in gastric cancer. *BioMed Research International*.

[44] Van den Bossche J., O'Neill L. A., Menon D. (2017). Macrophage Immunometabolism: where are we (going)?. *Trends in Immunology*.

[45] Zhang Q., Lou Y., Bai X. L., Liang T. B. (2018). Immunometabolism: a novel perspective of liver cancer microenvironment and its influence on tumor progression. *World Journal of Gastroenterology*.

[46] Dyck L., Lynch L. (2018). Cancer, obesity and immunometabolism - connecting the dots. *Cancer Letters*.

[47] Mazumdar C., Driggers E. M., Turka L. A. (2020). The untapped opportunity and challenge of immunometabolism: a new paradigm for drug discovery. *Cell Metabolism*.

[48] Newman A. M., Liu C. L., Green M. R. (2015). Robust enumeration of cell subsets from tissue expression profiles. *Nature Methods*.

[49] Li B., Severson E., Pignon J. C. (2016). Comprehensive analyses of tumor immunity: implications for cancer immunotherapy. *Genome Biology*.

[50] Alizadeh A. A., Aranda V., Bardelli A. (2015). Toward understanding and exploiting tumor heterogeneity. *Nature Medicine*.

[51] Kim N., Kim H. K., Lee K. (2020). Single-cell RNA sequencing demonstrates the molecular and cellular reprogramming of metastatic lung adenocarcinoma. *Nature Communications*.

[52] Cheng J., Zhang J., Wu Z., Sun X. (2021). Inferring microenvironmental regulation of gene expression from single-cell RNA sequencing data using scMLnet with an application to COVID-19. *Briefings in Bioinformatics*.

