# Supplementary material for: Expression Analysis of Ligand-Receptor Pairs Identifies Cell-to-Cell Crosstalk between Macrophages and Tumor Cells in Lung Adenocarcinoma
Source: J Immunol Res. 2022 Sep 22;2022:9589895. doi: 10.1155/2022/9589895 (PMC9553453; doi:10.1155/2022/9589895)

Supplement Figure 1:

A. The integration of single-cell data with Harmony shows the sample corresponding cohort (red cluster: samples from E-MTAB-6149; green cluster: samples from E-MTAB-6653; blue cluster: samples from previous literatures)

B. Three scRNA-seq are well integrated in the first 2 dimensions after Harmony

C. Overview distribution of the 159,219 single cells from 18 lung adenocarcinoma samples and 7 normal tissue samples (red cluster: normal samples; turquoise cluster: tumor samples)


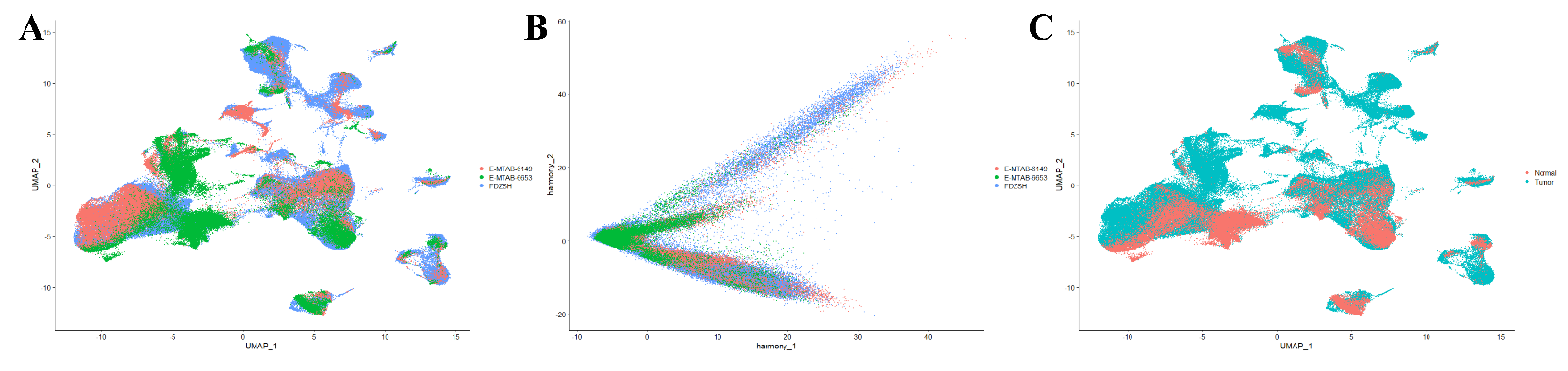


Supplement Figure 2: Expression of the cell typing marker genes for identifying tumor cells, alveolar cells and macrophages


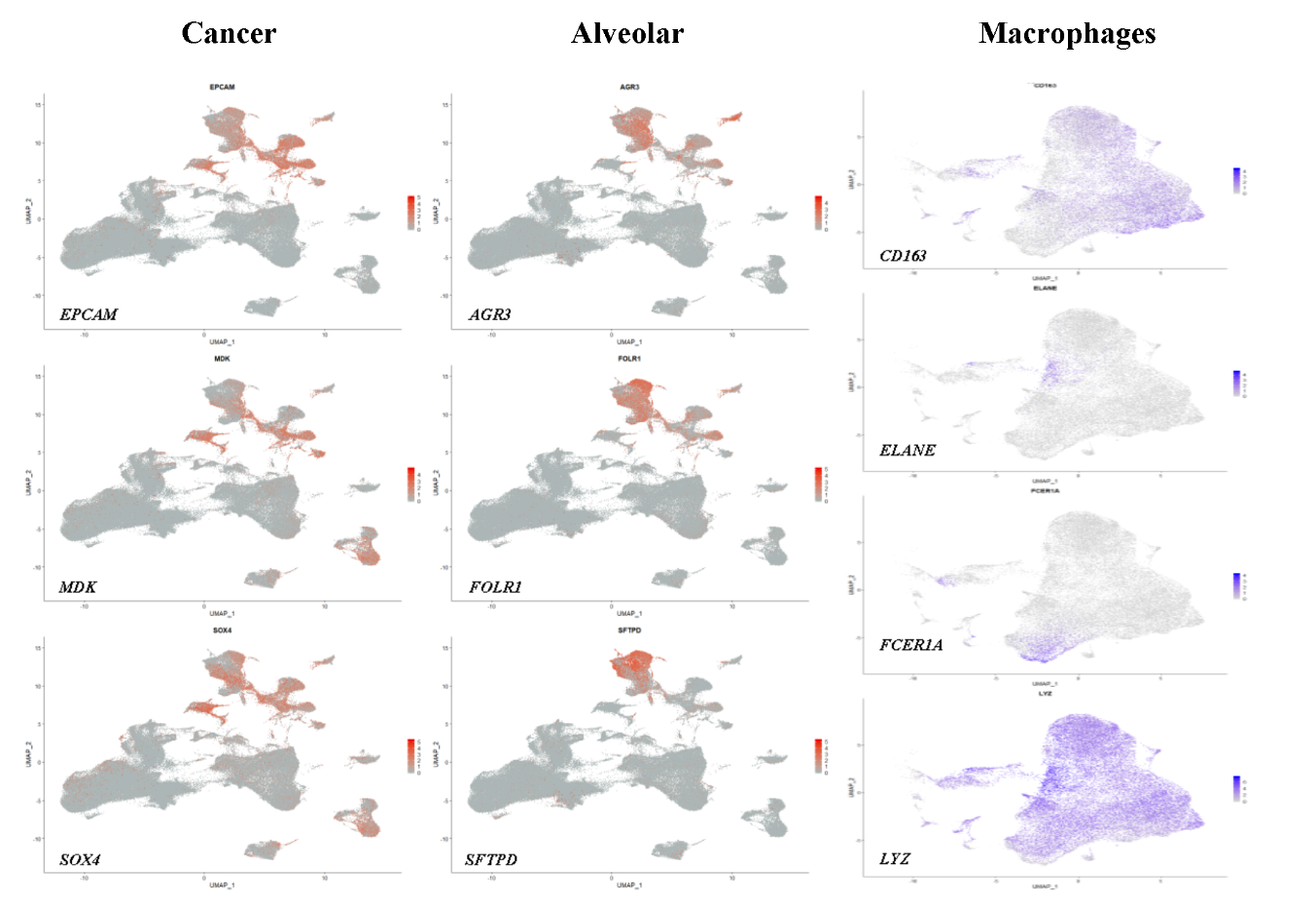


Supplement Figure 3:

A. Dot plot of the expression of marker genes for cell subtypes

B. Dot plot of the expression of marker genes for macrophages


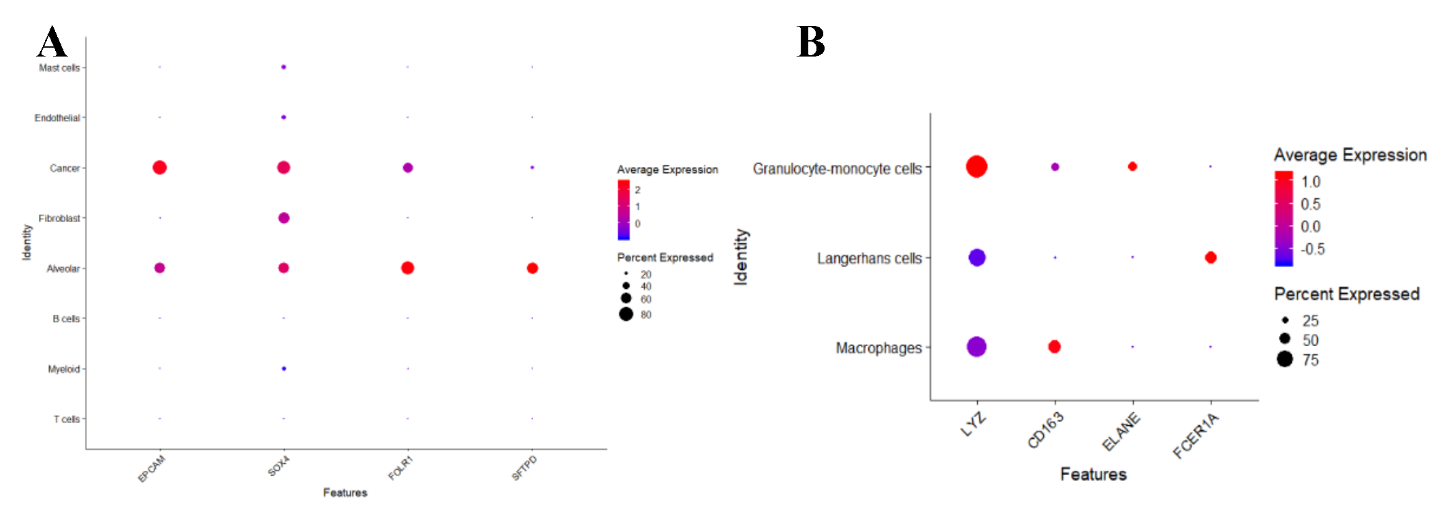


Supplement Figure 5

A. GO analysis for selected ligand-receptor genes in the crosstalk from macrophages to lung adenocarcinoma cells

B. GO analysis for selected ligand-receptor genes in the crosstalk from lung adenocarcinoma cells to macrophages


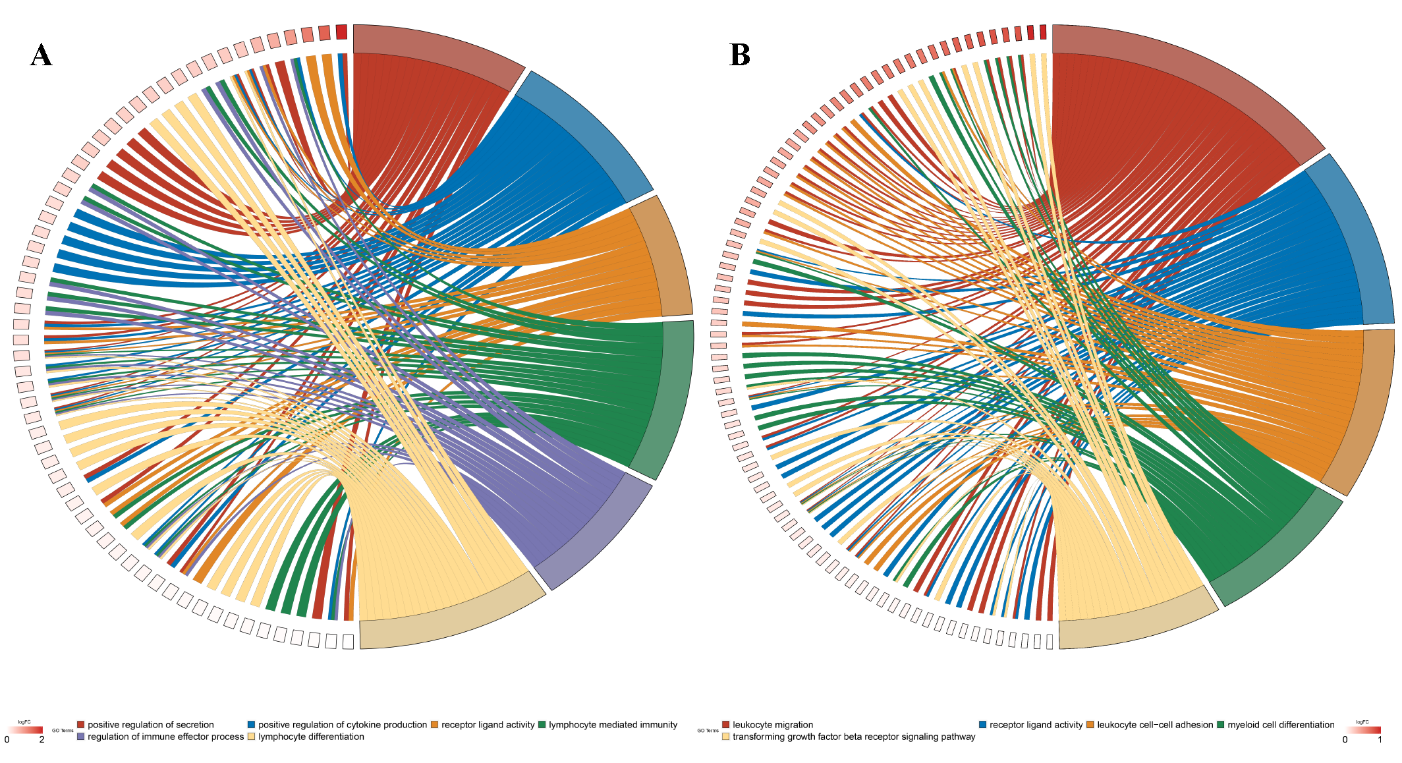


Supplement Figure 4

A. Heatmap of gene expression in the Hallmark *TGF-β* signaling pathway stratified by cell types in the scRNA-seq

B. Heatmap of gene expression in the KEGG allograft rejection signaling pathway stratified by cell types in the scRNA-seq

C. Heatmap of gene expression in the KEGG antigen processing and presentation signaling pathway stratified by cell types in the scRNA-seq


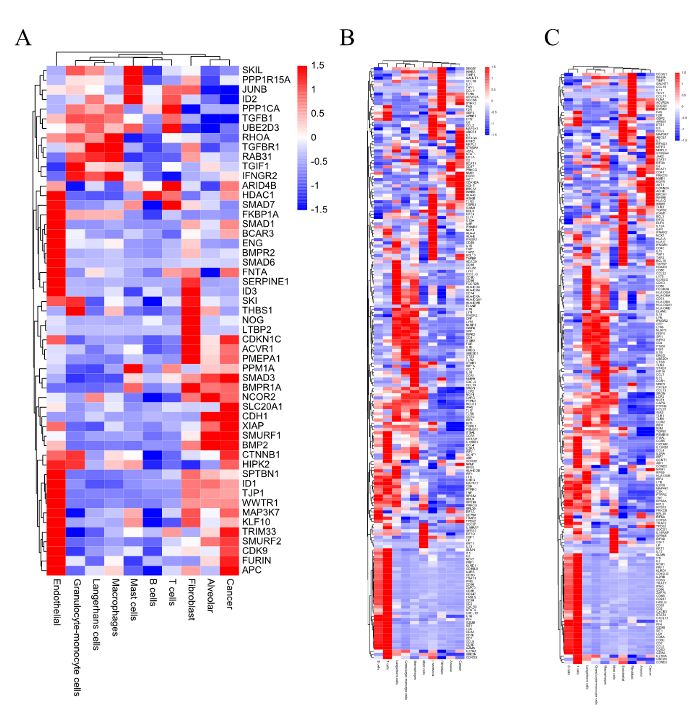


Supplement Figure 6: Identified and sorted the key cell marker genes in normal epithelial cells, lung adenocarcinoma cells and macrophages by flow cytometry

A-B. *FOLR1*+/*EPCAM*- cells accounted for larger proportions than *FOLR1*-/*EPCAM*+ in normal lung samples (0.30% vs 1.95%, 0.19 vs 1.32%) (X axis: PE-conjugated mouse anti-human *FOLR1*, Y axis: Alexa 647-conjugated mouse anti-human *EPCAM*).

C-D. *FOLR1*-/*EPCAM*+ cells accounted for larger proportions than *FOLR1*+/*EPCAM*- in lung adenocarcinoma samples (10.4% vs 2.03%, 17.1 vs 1.47%) (X axis: PE-conjugated mouse anti-human *FOLR1*, Y axis: Alexa 647-conjugated mouse anti-human *EPCAM*)

E-F. *CD163*+ cells (macrophages) accounted for 2.66% and 3.86% in normal lung samples (X axis: Alexa 647-conjugated mouse anti-human *CD163*)

G-H. *CD163*+ cells (macrophages) accounted for 6.86% and 6.65% in lung adenocarcinoma samples (X axis: Alexa 647-conjugated mouse anti-human *CD163*)


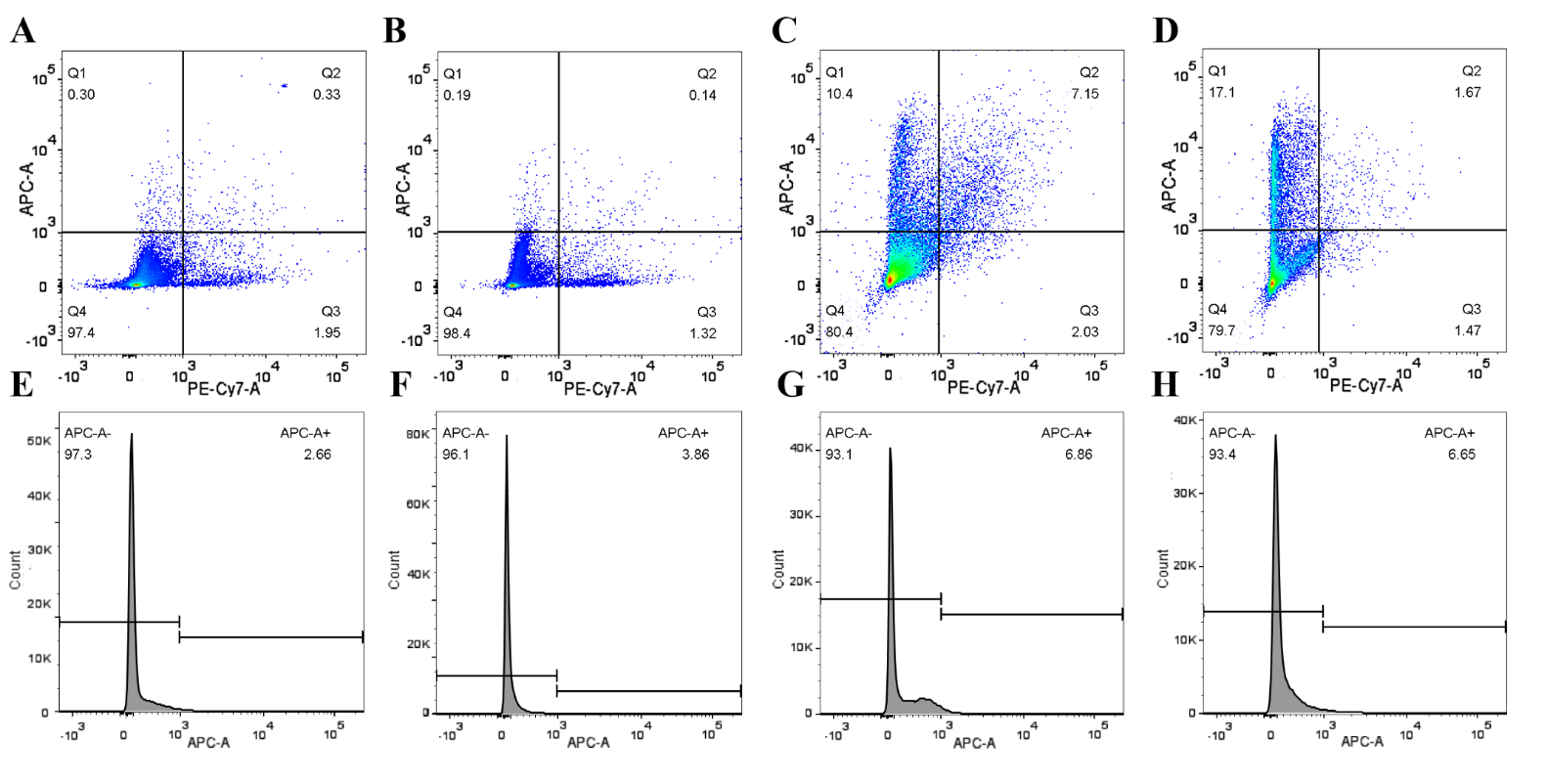


Supplement Figure 7: Associations of the expressions of selected ligand or receptor genes with macrophage infiltrating in lung adenocarcinoma of TCGA cohort by TIMER database

A. *TGFB1*(*P* < 0.05, Spearman’s ρ = 0.261)

B. *ENG* (*P* < 0.05, Spearman’s ρ =0.293)

C. *B2M* (*P* < 0.05, Spearman’s ρ = 0.175)

D. *HLA-F* (*P* > 0.05, Spearman’s ρ = -0.082)

E. *SELPLG* (*P* < 0.05, Spearman’s ρ = 0.321)

F. *ITGB2* (*P* < 0.05, Spearman’s ρ = 0.293)

G. *TGM2* (*P* < 0.05, Spearman’s ρ = 0.273)

H. *AGRP* (*P* < 0.05, Spearman’s ρ = 0.163)

I. *PTPRS* (*P* < 0.05, Spearman’s ρ = 0.169)

J. *CD4* (*P* < 0.05, Spearman’s ρ = 0.43)


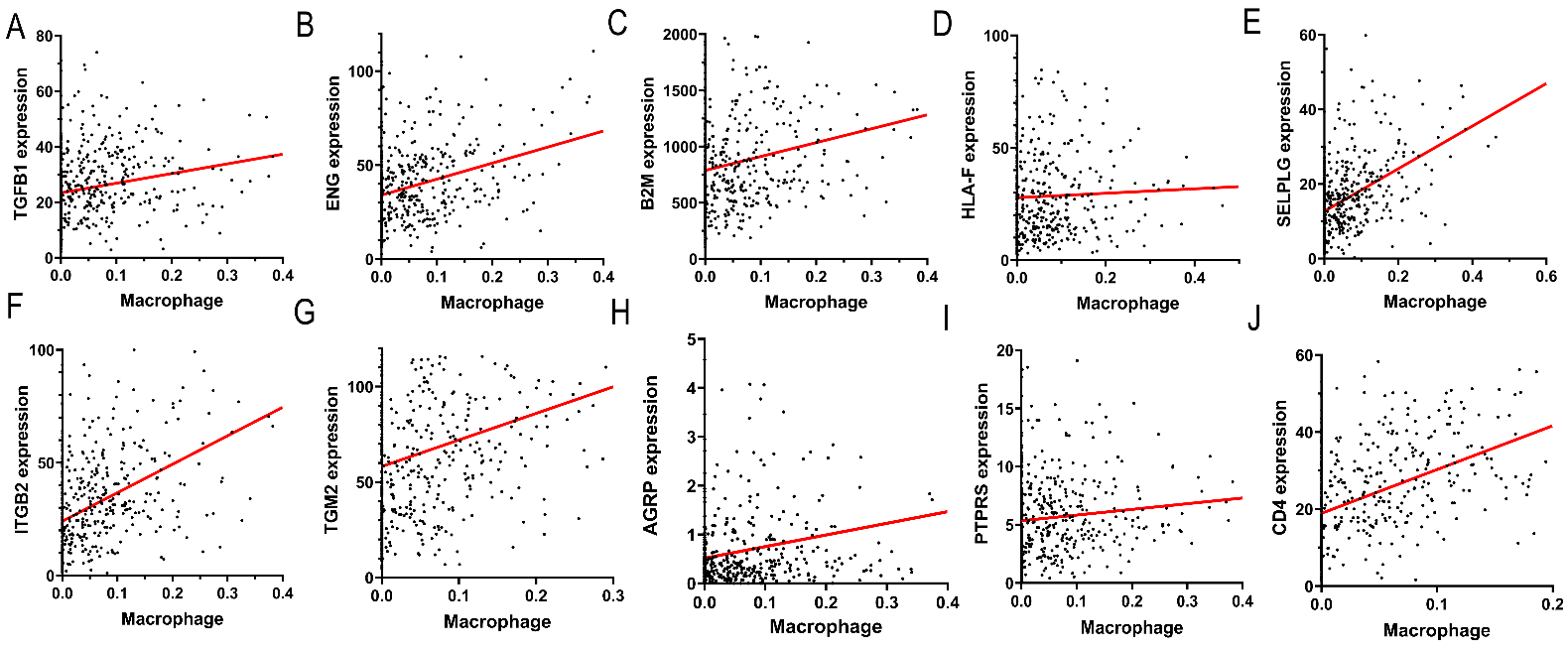


Supplement Figure 8: Validations of the expression changes of top ligand or receptor genes in lung adenocarcinoma cells and tumor-associated macrophages

A. The mRNA relative expressions level of *TGFB1*, *ENG*, *TGM2*, *TBXA2R*, *HSPG2* and *PTPRS* were significantly increased in lung adenocarcinoma cells than normal epithelial cells

B. The mRNA relative expressions of *TGFB1*, *ENG*, *B2M*, *HLA-F*, *SELPLG* and *ITGB2* were significantly increased in tumor-associated macrophages than macrophages


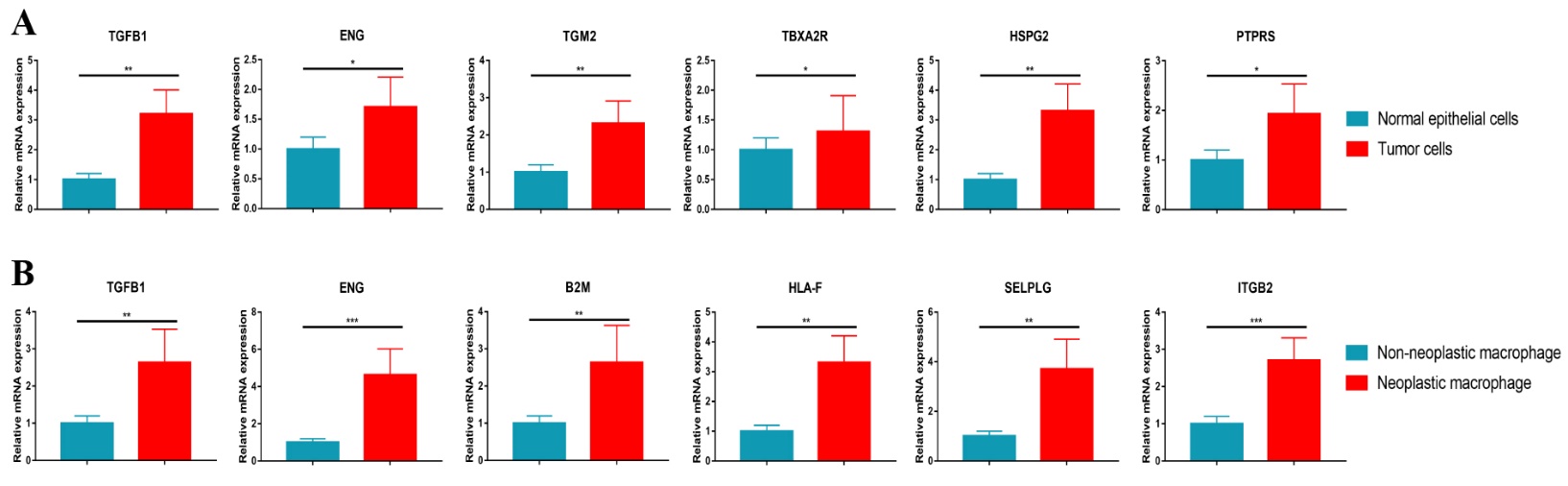

Supplement: Supplementary Materials — Supplement Figure 1: A. The integration of single-cell data with Harmony shows the sample corresponding cohort (red cluster: samples from E-MTAB-6149; green cluster: samples from E-MTAB-6653; blue cluster: samples from previous literatures). B. Three scRNA-seq are well integrated in the first 2 dimensions after Harmony. C. Overview distribution of the 159,219 single cells from 18 lung adenocarcinoma samples and 7 normal tissue samples (red cluster: normal samples; turquoise cluster: tumor samples). Supplement Figure 2: Expression of the cell typing marker genes for identifying tumor cells, alveolar cells, and macrophages. Supplement Figure 3: A. Dot plot of the expression of marker genes for cell subtypes. B. Dot plot of the expression of marker genes for macrophages. Supplement Figure 4. A. Heatmap of gene expression in the Hallmark TGF-β signaling pathway stratified by cell types in the scRNA-seq. B. Heatmap of gene expression in the KEGG allograft rejection signaling pathway stratified by cell types in the scRNA-seq. C. Heatmap of gene expression in the KEGG antigen processing and presentation signaling pathway stratified by cell types in the scRNA-seq. Supplement Figure 5. A. GO analysis for selected ligand-receptor genes in the crosstalk from macrophages to lung adenocarcinoma cells. B. GO analysis for selected ligand-receptor genes in the crosstalk from lung adenocarcinoma cells to macrophages. Supplement Figure 6: Identified and sorted the key cell marker genes in normal epithelial cells, lung adenocarcinoma cells, and macrophages by flow cytometry. A, B. FOLR1+/EPCAM- cells accounted for larger proportions than FOLR1-/EPCAM+ in normal lung samples (0.30% vs 1.95%, 0.19 vs 1.32%) (X-axis: PE-conjugated mouse antihuman FOLR1, Y-axis: Alexa 647-conjugated mouse antihuman EPCAM). C, D. FOLR1-/EPCAM+ cells accounted for larger proportions than FOLR1+/EPCAM- in lung adenocarcinoma samples (10.4% vs 2.03%, 17.1 vs 1.47%) (X-axis: PE-conjugated mouse antihuman FOLR1 [file 9589895.f1.zip › Supplement Figure V1.docx]
