# Supplementary material for: Expression Analysis of Ligand-Receptor Pairs Identifies Cell-to-Cell Crosstalk between Macrophages and Tumor Cells in Lung Adenocarcinoma
Source: J Immunol Res. 2022 Sep 22;2022:9589895. doi: 10.1155/2022/9589895 (PMC9553453; doi:10.1155/2022/9589895)
Supplement: Supplementary Materials — Supplement Figure 1: A. The integration of single-cell data with Harmony shows the sample corresponding cohort (red cluster: samples from E-MTAB-6149; green cluster: samples from E-MTAB-6653; blue cluster: samples from previous literatures). B. Three scRNA-seq are well integrated in the first 2 dimensions after Harmony. C. Overview distribution of the 159,219 single cells from 18 lung adenocarcinoma samples and 7 normal tissue samples (red cluster: normal samples; turquoise cluster: tumor samples). Supplement Figure 2: Expression of the cell typing marker genes for identifying tumor cells, alveolar cells, and macrophages. Supplement Figure 3: A. Dot plot of the expression of marker genes for cell subtypes. B. Dot plot of the expression of marker genes for macrophages. Supplement Figure 4. A. Heatmap of gene expression in the Hallmark TGF-β signaling pathway stratified by cell types in the scRNA-seq. B. Heatmap of gene expression in the KEGG allograft rejection signaling pathway stratified by cell types in the scRNA-seq. C. Heatmap of gene expression in the KEGG antigen processing and presentation signaling pathway stratified by cell types in the scRNA-seq. Supplement Figure 5. A. GO analysis for selected ligand-receptor genes in the crosstalk from macrophages to lung adenocarcinoma cells. B. GO analysis for selected ligand-receptor genes in the crosstalk from lung adenocarcinoma cells to macrophages. Supplement Figure 6: Identified and sorted the key cell marker genes in normal epithelial cells, lung adenocarcinoma cells, and macrophages by flow cytometry. A, B. FOLR1+/EPCAM- cells accounted for larger proportions than FOLR1-/EPCAM+ in normal lung samples (0.30% vs 1.95%, 0.19 vs 1.32%) (X-axis: PE-conjugated mouse antihuman FOLR1, Y-axis: Alexa 647-conjugated mouse antihuman EPCAM). C, D. FOLR1-/EPCAM+ cells accounted for larger proportions than FOLR1+/EPCAM- in lung adenocarcinoma samples (10.4% vs 2.03%, 17.1 vs 1.47%) (X-axis: PE-conjugated mouse antihuman FOLR1 [file 9589895.f1.zip › Supplement Methods (1).docx]

**Supplement Methods**

**The detailed methods of 10 × scRNA-seq and data preprocessing**

**Datasets**

Nine patients with primary lung cancer who received surgical resection were enrolled in this study. Post-operative pathological report confirmed the diagnosis of lung adenocarcinoma. Data were obtained from previous literature[1].

**Preparations of single cell suspension**

The process and methods of preparations of single cell suspension could be found in previous literature[1].

**Single cell RNA sequencing (scRNA-Seq)**

The process and methods of scRNA-seq could be found in previous literature[1].

**scRNA-Seq data preprocessing**

In this study, we used the Cell Ranger software pipeline (version 3.0) provided by 10×Genomics for demultiplex cellular barcodes, map reads to the genome, align transcriptomes using the STAR aligner and down-sample reads. A data matrix of gene expression (counts) versus single cells of all samples was produced. We processed the unique molecular identifier (UMI) count matrix via the Seurat package in R. We filtered out single cells of UMI/gene numbers with the threshold of mean values ± 2-fold of standard deviations for the purpose of removing low quality cells and multiplet captures. Furthermore, we excluded low quality cells which more than 10% counts belonged to mitochondrial genes, together with presentation of the distribution of cells by the fraction of mitochondrial genes expression. After the above calculation, single cell versus gene matrix was obtained.
