# Supplementary material for: Expression Analysis of Ligand-Receptor Pairs Identifies Cell-to-Cell Crosstalk between Macrophages and Tumor Cells in Lung Adenocarcinoma
Source: J Immunol Res. 2022 Sep 22;2022:9589895. doi: 10.1155/2022/9589895 (PMC9553453; doi:10.1155/2022/9589895)
Supplement: Supplementary Materials — Supplement Figure 1: A. The integration of single-cell data with Harmony shows the sample corresponding cohort (red cluster: samples from E-MTAB-6149; green cluster: samples from E-MTAB-6653; blue cluster: samples from previous literatures). B. Three scRNA-seq are well integrated in the first 2 dimensions after Harmony. C. Overview distribution of the 159,219 single cells from 18 lung adenocarcinoma samples and 7 normal tissue samples (red cluster: normal samples; turquoise cluster: tumor samples). Supplement Figure 2: Expression of the cell typing marker genes for identifying tumor cells, alveolar cells, and macrophages. Supplement Figure 3: A. Dot plot of the expression of marker genes for cell subtypes. B. Dot plot of the expression of marker genes for macrophages. Supplement Figure 4. A. Heatmap of gene expression in the Hallmark TGF-β signaling pathway stratified by cell types in the scRNA-seq. B. Heatmap of gene expression in the KEGG allograft rejection signaling pathway stratified by cell types in the scRNA-seq. C. Heatmap of gene expression in the KEGG antigen processing and presentation signaling pathway stratified by cell types in the scRNA-seq. Supplement Figure 5. A. GO analysis for selected ligand-receptor genes in the crosstalk from macrophages to lung adenocarcinoma cells. B. GO analysis for selected ligand-receptor genes in the crosstalk from lung adenocarcinoma cells to macrophages. Supplement Figure 6: Identified and sorted the key cell marker genes in normal epithelial cells, lung adenocarcinoma cells, and macrophages by flow cytometry. A, B. FOLR1+/EPCAM- cells accounted for larger proportions than FOLR1-/EPCAM+ in normal lung samples (0.30% vs 1.95%, 0.19 vs 1.32%) (X-axis: PE-conjugated mouse antihuman FOLR1, Y-axis: Alexa 647-conjugated mouse antihuman EPCAM). C, D. FOLR1-/EPCAM+ cells accounted for larger proportions than FOLR1+/EPCAM- in lung adenocarcinoma samples (10.4% vs 2.03%, 17.1 vs 1.47%) (X-axis: PE-conjugated mouse antihuman FOLR1 [file 9589895.f1.zip › Supplement Table 1 and Table 2 (1).docx]

Supplement Table 1 Characteristics of the 21 LUAD patients included in this study for scRNA-seq analysis

| Patient | Cell | Epithelial Cell | Macrophage | Age | Sex | Stage | Location | Mutation | Dataset |
| --- | --- | --- | --- | --- | --- | --- | --- | --- | --- |
| P1 | 269 | 63 | 82 | 85-90 | Male | - | RUL | - | ArrayExpress |
| **P2*** | 12998 | 1956 | 2643 | 65-70 | Male | IIIB | RUL | - | ArrayExpress |
| **P3*** | 16518 | 3031 | 2094 | 60-65 | Female | IIB | LUL | - | ArrayExpress |
| P4 | 5908 | 481 | 2298 | 60-65 | Male | - | LUL | - | ArrayExpress |
| **P5*** | 20232 | 777 | 1276 | 60-65 | Male | IIIA | LUL | - | ArrayExpress |
| P6 | 7848 | 237 | 896 | 60-65 | Male | - | LUL | - | ArrayExpress |
| P7 | 8063 | 897 | 3487 | 50-55 | Female | - | RUL | - | ArrayExpress |
| P8 | 6588 | 1298 | 676 | 60-65 | Female | IB | RUL | EGFR | Previous literature |
| P9 | 4549 | 1523 | 792 | 55-60 | Male | IB | LLL | EGFR | Previous literature |
| P10 | 5695 | 2708 | 896 | 70-75 | Male | IB | LLL | EGFR | Previous literature |
| P11 | 4212 | 709 | 369 | 65-70 | Female | IB | RLL | EGFR | Previous literature |
| P12 | 5568 | 1555 | 551 | 70-75 | Male | IB | LUL | EGFR | Previous literature |
| P13 | 9001 | 4606 | 1393 | 75-80 | Male | IA | LUL | EGFR | Previous literature |
| P14 | 5647 | 1410 | 1806 | 60-65 | Female | IB | RUL | EGFR | Previous literature |
| P15 | 5458 | 693 | 1315 | 55-60 | Female | IB | RML | EGFR | Previous literature |
| P16 | 6950 | 180 | 636 | 60-65 | Male | IA | LUL | EGFR | Previous literature |
| P17 | 7224 | 600 | 1368 | 55-60 | Female | IA | RUL | HER-2 | Previous literature |
| P18 | 6827 | 4018 | 1651 | 50-55 | Female | IIB | LLL | EGFR | Previous literature |
| P19 | 7399 | 4259 | 1071 | 65-70 | Female | IB | RML | EGFR | Previous literature |
| P20 | 7871 | 1360 | 3103 | 70-75 | Male | IIA | RUL | EGFR | Previous literature |
| P21 | 4394 | 599 | 1117 | 45-50 | Female | IIIA | RUL | EGFR | Previous literature |

*The patients provided the tumor and corresponding normal samples in our study.

RUL: Right upper lobe

RLL: Right lower lobe

RML: Light middle lobe

LLL: Left lower lobe

LUL: Left upper lobe

Data of selected patients (P8 to P21) were obtained from previous literature[1].

|  | GEO cohort-1 | GEO cohort-2 | TCGA cohort |
| --- | --- | --- | --- |
| Subset | GSE30219, GSE31210,  GSE50081, GSE37745 | GSE68465 | / |
| Platform | HG-U133 Plus 2.0 (GPL570) | HG-U133A (GPL96) | RNA-seq |
| Patient Number | 544 | 443 | 493 |
| Gender |  |  |  |
| Male | 282 (51.84) | 223 (50.34) | 226 (45.84) |
| Female | 262 (48.16) | 220 (49.66) | 267 (54.16) |
| Age | 62.67 ± 9.33 | 64.42 ± 10.09 | 65.23 ± 10.05 |
| Stage |  |  |  |
| I-II stage | 526 (96.69) | 372 (83.97) | 388 (78.70) |
| III-IV stage | 18 (3.31) | 68 (15.35) | 105 (21.30) |
| Unknown | 0 | 3 (0.68) | 0 |

Supplement Table 2: Baseline characteristics of enrolled patient cohorts from GEO and TCGA databases.

GEO: Gene Expression Omnibus

TCGA: The Cancer Genome Atlas
