# Supplementary material for: Expression Analysis of Ligand-Receptor Pairs Identifies Cell-to-Cell Crosstalk between Macrophages and Tumor Cells in Lung Adenocarcinoma
Source: J Immunol Res. 2022 Sep 22;2022:9589895. doi: 10.1155/2022/9589895 (PMC9553453; doi:10.1155/2022/9589895)
Supplement: Supplementary Materials — Supplement Figure 1: A. The integration of single-cell data with Harmony shows the sample corresponding cohort (red cluster: samples from E-MTAB-6149; green cluster: samples from E-MTAB-6653; blue cluster: samples from previous literatures). B. Three scRNA-seq are well integrated in the first 2 dimensions after Harmony. C. Overview distribution of the 159,219 single cells from 18 lung adenocarcinoma samples and 7 normal tissue samples (red cluster: normal samples; turquoise cluster: tumor samples). Supplement Figure 2: Expression of the cell typing marker genes for identifying tumor cells, alveolar cells, and macrophages. Supplement Figure 3: A. Dot plot of the expression of marker genes for cell subtypes. B. Dot plot of the expression of marker genes for macrophages. Supplement Figure 4. A. Heatmap of gene expression in the Hallmark TGF-β signaling pathway stratified by cell types in the scRNA-seq. B. Heatmap of gene expression in the KEGG allograft rejection signaling pathway stratified by cell types in the scRNA-seq. C. Heatmap of gene expression in the KEGG antigen processing and presentation signaling pathway stratified by cell types in the scRNA-seq. Supplement Figure 5. A. GO analysis for selected ligand-receptor genes in the crosstalk from macrophages to lung adenocarcinoma cells. B. GO analysis for selected ligand-receptor genes in the crosstalk from lung adenocarcinoma cells to macrophages. Supplement Figure 6: Identified and sorted the key cell marker genes in normal epithelial cells, lung adenocarcinoma cells, and macrophages by flow cytometry. A, B. FOLR1+/EPCAM- cells accounted for larger proportions than FOLR1-/EPCAM+ in normal lung samples (0.30% vs 1.95%, 0.19 vs 1.32%) (X-axis: PE-conjugated mouse antihuman FOLR1, Y-axis: Alexa 647-conjugated mouse antihuman EPCAM). C, D. FOLR1-/EPCAM+ cells accounted for larger proportions than FOLR1+/EPCAM- in lung adenocarcinoma samples (10.4% vs 2.03%, 17.1 vs 1.47%) (X-axis: PE-conjugated mouse antihuman FOLR1 [file 9589895.f1.zip › Supplement Table 3 (1).docx]

| Significant autocrine ligand-receptor gene pairs of tumor cells in lung adenocarcinoma | | | |
| --- | --- | --- | --- |
| Expression | Ligand | Receptor | Correlation efficient |
| Up | TGFB1 | ENG | 0.782291997 |
| Up | TGM2 | TBXA2R | 0.696744505 |
| Up | HSPG2 | PTPRS | 0.681603764 |
| Up | BMP5 | ACVR2A | 0.64877485 |
| Up | HLA-G | KIR2DL4 | 0.639582074 |
| Up | CD70 | CD27 | 0.626664609 |
| Up | BMP5 | BMPR1A | 0.611340298 |
| Up | SEMA3F | PLXNA1 | 0.582042669 |
| Up | APP | SLC45A3 | 0.552056713 |
| Up | DLL3 | NOTCH3 | 0.538743888 |
| Up | CALCA | RAMP2 | 0.530461815 |
| Up | L1CAM | EPHB2 | 0.494805302 |
| Up | PI3 | PLD2 | 0.491647649 |
| Up | LTBP1 | ITGB5 | 0.485227335 |
| Up | PSEN1 | NOTCH3 | 0.484631589 |
| Up | PSEN1 | NOTCH2 | 0.483288936 |
| Up | ADAM9 | ITGB1 | 0.463637861 |
| Up | APP | GPC1 | 0.456204272 |
| Up | APP | TNFRSF21 | 0.454145865 |
| Up | TGFB1 | CXCR4 | 0.441303822 |
| Up | TGFB3 | ENG | 0.425720244 |
| Up | HRAS | GRIN2D | 0.424422728 |
| Up | LTBP3 | ITGB5 | 0.42026918 |
| Up | HLA-G | KLRD1 | 0.417124299 |
| Up | FGG | ITGB1 | 0.416844053 |
| Up | LAMA4 | ITGB1 | 0.415183192 |
| Up | DLL3 | NOTCH4 | 0.402418087 |
| Up | SLIT1 | GPC1 | 0.399588678 |
| Up | EFNA5 | EPHA4 | 0.395866487 |
| Up | GPI | AMFR | 0.387866111 |
| Up | TNF | LTBR | 0.381628987 |
| Up | CXCL12 | CXCR4 | 0.372052288 |
| Up | JAG2 | NOTCH3 | 0.363873675 |
| Up | FGA | ITGB1 | 0.359848429 |
| Up | KISS1 | MMP24 | 0.356011374 |
| Up | TGM2 | ITGB1 | 0.350621637 |
| Up | FGB | ITGB1 | 0.347985575 |
| Up | VEGFC | KDR | 0.332792655 |
| Up | DLL3 | NOTCH2 | 0.316817535 |
| Up | SORBS1 | ITGB5 | 0.311837724 |
| Up | NID1 | ITGB1 | 0.308996519 |
| Up | ACE | AGTR2 | 0.304601232 |
| Up | EFNB3 | EPHB2 | 0.303661135 |
| Up | VCAN | ITGB1 | 0.301627618 |
| Down | B2M | HLA-F | 0.737035186 |
| Down | SELPLG | ITGB2 | 0.711098862 |
| Down | IL1RN | IL1RL2 | 0.683352456 |
| Down | ICAM3 | ITGAL | 0.681819987 |
| Down | SERPING1 | LRP1 | 0.661577101 |
| Down | B2M | HFE | 0.615843071 |
| Down | VEGFA | SIRPA | 0.596825565 |
| Down | CXCL13 | CXCR3 | 0.591886506 |
| Down | SELPLG | ITGAM | 0.5643738 |
| Down | GAS6 | AXL | 0.560457333 |
| Down | ADAM15 | ITGB3 | 0.558664331 |
| Down | C1QB | LRP1 | 0.548199683 |
| Down | VEGFA | NRP2 | 0.546516019 |
| Down | HLA-C | LILRB1 | 0.539731456 |
| Down | HLA-B | LILRB1 | 0.53321349 |
| Down | CSF2 | CSF2RA | 0.525271613 |
| Down | C1QA | CR1 | 0.519893695 |
| Down | ALOX5AP | ALOX5 | 0.518256631 |
| Down | PDGFB | LRP1 | 0.515743968 |
| Down | APOE | LRP1 | 0.510011788 |
| Down | COL4A4 | CD47 | 0.501501523 |
| Down | ADAM15 | ITGA5 | 0.498172071 |
| Down | RARRES2 | CMKLR1 | 0.49106797 |
| Down | MMP9 | ITGB2 | 0.487649859 |
| Down | EFNB1 | EPHB3 | 0.48156653 |
| Down | ICAM2 | ITGAL | 0.48054411 |
| Down | ICAM2 | ITGB2 | 0.474941148 |
| Down | ICAM3 | ITGB2 | 0.472054821 |
| Down | C3 | CD81 | 0.46748593 |
| Down | ICAM1 | ITGB2 | 0.463651578 |
| Down | IL10 | IL10RB | 0.463295077 |
| Down | HLA-A | LILRB1 | 0.459338098 |
| Down | B2M | LILRB1 | 0.451201793 |
| Down | FN1 | ITGAV | 0.44063901 |
| Down | SELPLG | SELL | 0.438111533 |
| Down | SERPINE2 | LRP1 | 0.422492811 |
| Down | CXCL9 | CXCR3 | 0.42055043 |
| Down | CYR61 | ITGB2 | 0.405667436 |
| Down | MMP9 | LRP1 | 0.401450711 |
| Down | LAMC2 | ITGA2 | 0.396491812 |
| Down | EFNB1 | ERBB2 | 0.39438279 |
| Down | TNFSF14 | TNFRSF14 | 0.379405386 |
| Down | LYZ | ITGAL | 0.379316636 |
| Down | LAMC2 | ITGA3 | 0.377187856 |
| Down | PTN | PTPRB | 0.374377748 |
| Down | MMP9 | ITGAM | 0.374135803 |
| Down | COL4A4 | ITGA1 | 0.373191003 |
| Down | COL4A5 | ITGA1 | 0.369854245 |
| Down | CCL2 | CCR10 | 0.366490069 |
| Down | CCL2 | CCR1 | 0.364215688 |
| Down | CYR61 | ITGA5 | 0.358108517 |
| Down | A2M | LRP1 | 0.354796763 |
| Down | CSF1 | CSF1R | 0.33554002 |
| Down | CCL23 | CCR1 | 0.333771459 |
| Down | CXCL13 | CCR10 | 0.332876157 |
| Down | C3 | ITGAM | 0.31485055 |
| Down | PDGFA | PDGFRA | 0.313619558 |
| Down | MMP9 | CD44 | 0.310182334 |
| Down | CYR61 | ITGB3 | 0.308945776 |
| Down | ICAM1 | ITGAX | 0.30697941 |
| Down | IL10 | IL10RA | 0.305592055 |
| Down | THBS1 | CD36 | 0.30442464 |
| Down | ICAM4 | ITGAM | 0.302192419 |

| Significant autocrine ligand-receptor gene pairs of macropahges in lung adenocarcinoma | | | |
| --- | --- | --- | --- |
| Expression | Ligand | Receptor | Correlation Efficient |
| Up | TGFB1 | ENG | 0.782291997 |
| Up | B2M | HLA-F | 0.737035186 |
| Up | SELPLG | ITGB2 | 0.711098862 |
| Up | SERPING1 | LRP1 | 0.661577101 |
| Up | AGRP | SDC3 | 0.641189332 |
| Up | HLA-G | CD4 | 0.64088098 |
| Up | CCL4 | CCR1 | 0.627965424 |
| Up | MFGE8 | ITGB3 | 0.621635555 |
| Up | B2M | HFE | 0.615843071 |
| Up | CALR | HLA-F | 0.604491945 |
| Up | TGM2 | ITGB3 | 0.59913324 |
| Up | TGFB1 | ACVRL1 | 0.581101898 |
| Up | GNAI2 | C5AR1 | 0.57813836 |
| Up | SPINT1 | ST14 | 0.576309307 |
| Up | CCL8 | CCR1 | 0.569305877 |
| Up | PLAU | ITGA5 | 0.56902 |
| Up | SELPLG | ITGAM | 0.5643738 |
| Up | MDK | SDC3 | 0.561226416 |
| Up | GNAI2 | S1PR4 | 0.55263924 |
| Up | PSEN1 | NCSTN | 0.548541962 |
| Up | C1QB | LRP1 | 0.548199683 |
| Up | C1QA | CR1 | 0.519893695 |
| Up | ALOX5AP | ALOX5 | 0.518256631 |
| Up | PDGFB | LRP1 | 0.515743968 |
| Up | APOE | LRP1 | 0.510011788 |
| Up | TNFSF13 | TNFRSF14 | 0.504804508 |
| Up | GNAI2 | UNC5B | 0.491231141 |
| Up | APP | NCSTN | 0.488312285 |
| Up | MMP9 | ITGB2 | 0.487649859 |
| Up | PSEN1 | NOTCH2 | 0.483288936 |
| Up | ICAM2 | ITGAL | 0.48054411 |
| Up | ICAM2 | ITGB2 | 0.474941148 |
| Up | CALR | LRP1 | 0.465392263 |
| Up | ADAM9 | ITGB1 | 0.463637861 |
| Up | APP | TNFRSF21 | 0.454145865 |
| Up | GNAI2 | ADORA1 | 0.45071906 |
| Up | POMC | MC1R | 0.449724971 |
| Up | CAMP | FPR2 | 0.443029705 |
| Up | PSAP | LRP1 | 0.440895401 |
| Up | FN1 | ITGAV | 0.44063901 |
| Up | CCL7 | CCR1 | 0.430469864 |
| Up | ADAM9 | ITGAV | 0.429708365 |
| Up | PLAU | ITGB2 | 0.424324638 |
| Up | GNAI2 | IGF1R | 0.421364108 |
| Up | MMP9 | EPHB2 | 0.421356238 |
| Up | LTBP3 | ITGB5 | 0.42026918 |
| Up | EFNB1 | EPHB2 | 0.412134069 |
| Up | IL7 | IL7R | 0.411942717 |
| Up | PSAP | CELSR1 | 0.411563393 |
| Up | MMP9 | LRP1 | 0.401450711 |
| Up | SCT | VIPR2 | 0.398929056 |
| Up | FARP2 | PLXNA2 | 0.395211931 |
| Up | IL1A | IL1RAP | 0.394914072 |
| Up | SERPINE1 | LRP1 | 0.390842679 |
| Up | GPI | AMFR | 0.387866111 |
| Up | PLAU | IGF2R | 0.38738327 |
| Up | PLAU | ST14 | 0.385997482 |
| Up | FGF10 | FGFR1 | 0.383661048 |
| Up | LYZ | ITGAL | 0.379316636 |
| Up | APOE | LDLR | 0.377040463 |
| Up | MMP9 | ITGAM | 0.374135803 |
| Up | CXCL12 | ACKR3 | 0.369620245 |
| Up | SEMA3B | NRP2 | 0.369437046 |
| Up | FBN1 | ITGAV | 0.36713701 |
| Up | CCL2 | CCR1 | 0.364215688 |
| Up | PSEN1 | CD44 | 0.361035212 |
| Up | A2M | LRP1 | 0.354796763 |
| Up | COL6A1 | ITGB1 | 0.354177285 |
| Up | TGM2 | ITGB1 | 0.350621637 |
| Up | JAG1 | NOTCH1 | 0.348537447 |
| Up | TGFB2 | TGFBR2 | 0.347307259 |
| Up | PKM | CD44 | 0.343296578 |
| Up | CCL23 | CCR1 | 0.333771459 |
| Up | VEGFC | KDR | 0.332792655 |
| Up | APP | LRP1 | 0.329258593 |
| Up | PLAT | LRP1 | 0.323479344 |
| Up | PLAU | LRP1 | 0.323387003 |
| Up | APP | FPR2 | 0.317122515 |
| Up | CCL13 | CCR1 | 0.314737286 |
| Up | PLAT | ITGAM | 0.313833471 |
| Up | TSLP | IL7R | 0.312206087 |
| Up | MMP9 | CD44 | 0.310182334 |
| Up | APP | CD74 | 0.303344013 |
| Up | MDK | ITGB1 | 0.30263167 |
| Down | CCL19 | CCR7 | 0.72158787 |
| Down | IL1RN | IL1RL2 | 0.683352456 |
| Down | CCL19 | CXCR3 | 0.629123784 |
| Down | CD70 | CD27 | 0.626664609 |
| Down | CXCL13 | CXCR5 | 0.618890962 |
| Down | CXCL13 | CXCR3 | 0.591886506 |
| Down | GAL | GALR2 | 0.523881441 |
| Down | BTLA | CD247 | 0.49810051 |
| Down | TNFSF8 | TNFRSF8 | 0.495374347 |
| Down | IL1RN | IL1R2 | 0.479926955 |
| Down | WNT5A | FZD1 | 0.42667556 |
| Down | LTA | TNFRSF1B | 0.413504941 |
| Down | CSF2 | CSF3R | 0.40667892 |
| Down | CXCL10 | CXCR3 | 0.403565182 |
| Down | DLL3 | NOTCH4 | 0.402418087 |
| Down | IL1B | IL1R2 | 0.383798166 |
| Down | VEGFA | GPC1 | 0.374586691 |
| Down | COL4A4 | ITGA1 | 0.373191003 |
| Down | COL4A5 | ITGA1 | 0.369854245 |
| Down | KISS1 | KISS1R | 0.360274306 |
| Down | VEGFA | TYRO3 | 0.347629191 |
| Down | KITLG | EPOR | 0.347067488 |
| Down | FGA | PLAUR | 0.341059648 |
| Down | CALCA | RAMP1 | 0.337975142 |
| Down | CALCB | RAMP1 | 0.336933347 |

| Macrophages connect with tumor cells in lung adenocarcinoma | | | | |
| --- | --- | --- | --- | --- |
| Ligand | Receptor | Correlation Efficient | Ligand Prognostic P-value | Receptor Prognostic P-value |
| TGFB1 | ENG | 0.782291997 | 7.82E-10 | 0.025778654 |
| TGM2 | TBXA2R | 0.696744505 | 2.86E-05 | 4.93E-10 |
| AGRP | SDC3 | 0.641189332 | 3.16E-06 | 8.80E-07 |
| HLA-G | KIR2DL4 | 0.639582074 | 4.32E-08 | 1.95E-05 |
| GNAI2 | TBXA2R | 0.636322174 | 0.002289333 | 4.93E-10 |
| HLA-C | CD3D | 0.593689027 | 0.764894604 | 0.578927624 |
| MDK | SDC3 | 0.561226416 | 2.21E-12 | 8.80E-07 |
| APP | SLC45A3 | 0.552056713 | 4.74E-09 | 3.50E-08 |
| HLA-B | CD3D | 0.521517605 | 1.11E-05 | 0.578927624 |
| B2M | KLRD1 | 0.504663748 | 9.94E-05 | 0.006014286 |
| GNAI2 | PTPRU | 0.503109759 | 0.002289333 | 0.005470545 |
| HLA-A | CD3D | 0.49718114 | 0.312725737 | 0.578927624 |
| PSEN1 | NOTCH3 | 0.484631589 | 1.19E-07 | 7.83E-10 |
| PSEN1 | NOTCH2 | 0.483288936 | 1.19E-07 | 0.000388714 |
| HLA-C | CD3G | 0.479725709 | 0.764894604 | 0.669578429 |
| ADAM9 | ITGB1 | 0.463637861 | 0.462839791 | 0.000219547 |
| APP | GPC1 | 0.456204272 | 4.74E-09 | 2.75E-07 |
| VEGFB | TYRO3 | 0.455072047 | 0.000269962 | 0.00030095 |
| APP | TNFRSF21 | 0.454145865 | 4.74E-09 | 3.90E-10 |
| GNAI2 | ADORA1 | 0.45071906 | 0.002289333 | 2.49E-05 |
| TGFB1 | CXCR4 | 0.441303822 | 7.82E-10 | 0.003565299 |
| EFNB1 | EPHB6 | 0.426627388 | 3.25E-06 | 0.203427108 |
| GNAI2 | IGF1R | 0.421364108 | 0.002289333 | 2.63E-05 |
| MMP9 | EPHB2 | 0.421356238 | 0.000220315 | 4.13E-08 |
| LTBP3 | ITGB5 | 0.42026918 | 0.019276089 | 0.331821689 |
| HLA-G | KLRD1 | 0.417124299 | 4.32E-08 | 0.006014286 |
| JAG1 | NOTCH3 | 0.41620846 | 0.000985128 | 7.83E-10 |
| HLA-A | CD3G | 0.414522385 | 0.312725737 | 0.669578429 |
| EFNB1 | EPHB2 | 0.412134069 | 3.25E-06 | 4.13E-08 |
| FGF10 | FGFR2 | 0.410911722 | 6.39E-05 | 0.194734869 |
| SLIT1 | GPC1 | 0.399588678 | 3.95E-09 | 2.75E-07 |
| IL1A | IL1RAP | 0.394914072 | 0.519944723 | 0.038724389 |
| B2M | CD3D | 0.392614094 | 9.94E-05 | 0.578927624 |
| GPI | AMFR | 0.387866111 | 1.04E-14 | 0.350726511 |
| HLA-B | KLRD1 | 0.381008122 | 1.11E-05 | 0.006014286 |
| APOE | LDLR | 0.377040463 | 0.00020825 | 1.41E-06 |
| CXCL12 | CXCR4 | 0.372052288 | 9.73E-05 | 0.003565299 |
| IL1A | IL1R1 | 0.371936862 | 0.519944723 | 5.33E-06 |
| APOE | LRP5 | 0.355906802 | 0.00020825 | 4.05E-10 |
| COL6A1 | ITGB1 | 0.354177285 | 5.01E-10 | 0.000219547 |
| TGM2 | ITGB1 | 0.350621637 | 2.86E-05 | 0.000219547 |
| TGFB2 | TGFBR2 | 0.347307259 | 0.011850769 | 1.69E-05 |
| JAG1 | NOTCH4 | 0.342848933 | 0.000985128 | 0.046757153 |
| IL6 | F3 | 0.337440653 | 0.00041311 | 0.271563546 |
| VEGFC | KDR | 0.332792655 | 0.03820809 | 0.016661305 |
| DUSP18 | ITGB4 | 0.32629005 | 0.090705998 | 2.83E-10 |
| GNAI2 | ADRA2B | 0.319329488 | 0.002289333 | 4.05E-07 |
| GUCA2B | GUCY2D | 0.3155144 | 0.001312827 | 0.958918807 |
| ACE | AGTR2 | 0.304601232 | 0.001765639 | 0.247203462 |
| LPL | VLDLR | 0.303619162 | 8.68E-08 | 0.00286352 |
| MDK | ITGB1 | 0.30263167 | 2.21E-12 | 0.000219547 |
| HLA-B | CD3G | 0.301781982 | 1.11E-05 | 0.669578429 |

| Tumor cells connect with macrophages in lung adenocarcinoma | | | | |
| --- | --- | --- | --- | --- |
| Ligand | Receptor | Correlation Efficient | Ligand Prognostic P-value | Receptor Prognostic P-value |
| TGFB1 | ENG | 0.753086866 | 7.82E-10 | 0.025778654 |
| HSPG2 | PTPRS | 0.686329787 | 1.18E-05 | 0.000127696 |
| HLA-G | CD4 | 0.65112565 | 4.32E-08 | 0.000522346 |
| BMP5 | ACVR2A | 0.646339517 | 5.52E-06 | 0.000243781 |
| MFGE8 | ITGB3 | 0.632727553 | 4.94E-05 | 1.69E-05 |
| BMP5 | BMPR1A | 0.624070462 | 5.52E-06 | 1.08E-08 |
| CALR | HLA-F | 0.612560444 | 1.43E-09 | 2.63E-05 |
| SPINT1 | ST14 | 0.609906234 | 9.23E-11 | 6.30E-06 |
| TGM2 | ITGB3 | 0.609594831 | 2.86E-05 | 1.69E-05 |
| SEMA3F | PLXNA1 | 0.587387234 | 1.23E-09 | 2.12E-10 |
| CCL5 | CCR1 | 0.566265668 | 0.24823849 | 0.059261347 |
| PSEN1 | NCSTN | 0.56006697 | 1.19E-07 | 6.33E-06 |
| COL1A1 | ITGA5 | 0.557067325 | 7.27E-12 | 2.35E-08 |
| SEMA3F | PLXNA3 | 0.551304079 | 1.23E-09 | 5.48E-09 |
| SEMA3F | NRP2 | 0.52950276 | 1.23E-09 | 6.64E-10 |
| TGFB1 | ACVRL1 | 0.521148457 | 7.82E-10 | 0.005433518 |
| PSEN1 | NOTCH2 | 0.508488805 | 1.19E-07 | 0.000388714 |
| APP | NCSTN | 0.508475335 | 4.74E-09 | 6.33E-06 |
| PI3 | PLD2 | 0.508432829 | 1.07E-05 | 2.86E-07 |
| IL16 | KCNA3 | 0.503927701 | 1.33E-13 | 1.42E-05 |
| L1CAM | EPHB2 | 0.50096362 | 8.64E-07 | 4.13E-08 |
| LTBP1 | ITGB5 | 0.490978637 | 5.90E-09 | 0.331821689 |
| ADAM9 | ITGB1 | 0.481488382 | 0.462839791 | 0.000219547 |
| TGFB3 | ACVRL1 | 0.478018759 | 0.007412122 | 0.005433518 |
| UBA52 | NOTCH1 | 0.468588139 | 1.39E-05 | 0.392855035 |
| CTGF | LRP1 | 0.467519387 | 0.015761561 | 0.048201018 |
| L1CAM | ITGA5 | 0.456653536 | 8.64E-07 | 2.35E-08 |
| POMC | MC1R | 0.45349395 | 0.000151659 | 1.58E-06 |
| COL1A1 | CD44 | 0.453140475 | 7.27E-12 | 0.04031177 |
| APP | TNFRSF21 | 0.442338734 | 4.74E-09 | 3.90E-10 |
| CALR | LRP1 | 0.439671577 | 1.43E-09 | 0.048201018 |
| LY6G5C | TMEM8A | 0.436509449 | 0.224540369 | 0.000199632 |
| FGA | ITGAD | 0.43648097 | 0.000722062 | 1.10E-06 |
| HRAS | GRIN2D | 0.432648761 | 4.10E-08 | 4.81E-05 |
| LTBP3 | ITGB5 | 0.423855693 | 0.019276089 | 0.331821689 |
| TGFB3 | ENG | 0.41062542 | 0.007412122 | 0.025778654 |
| FARP2 | PLXNA2 | 0.408553391 | 7.24E-05 | 6.37E-14 |
| CXCL12 | ACKR3 | 0.407527108 | 9.73E-05 | 0.046718101 |
| LAMA4 | ITGB1 | 0.406306725 | 1.01E-12 | 0.000219547 |
| FGG | ITGB1 | 0.404249884 | 0.012949521 | 0.000219547 |
| GPI | AMFR | 0.396839898 | 1.04E-14 | 0.350726511 |
| PSEN1 | CD44 | 0.39647323 | 1.19E-07 | 0.04031177 |
| GDF11 | ACVR1B | 0.390626308 | 0.299319571 | 8.85E-06 |
| NTF3 | NTRK3 | 0.389228143 | 0.464135815 | 0.222780712 |
| FST | BMPR2 | 0.387770358 | 0.873518922 | 0.00023084 |
| ADAM9 | ITGAV | 0.387447085 | 0.462839791 | 0.055215536 |
| VEGFC | KDR | 0.379692014 | 0.03820809 | 0.016661305 |
| GZMB | IGF2R | 0.364204368 | 3.22E-07 | 0.000234553 |
| TNF | LTBR | 0.363857237 | 0.294690323 | 3.95E-09 |
| TSLP | IL7R | 0.363727735 | 2.18E-05 | 4.55E-07 |
| PKM | CD44 | 0.361007558 | 1.00E-13 | 0.04031177 |
| TGM2 | ITGB1 | 0.360173586 | 2.86E-05 | 0.000219547 |
| JAG2 | NOTCH1 | 0.358053171 | 0.002900525 | 0.392855035 |
| SEMA3B | NRP2 | 0.350699888 | 1.08E-05 | 6.64E-10 |
| EFNB3 | EPHB2 | 0.347108374 | 0.02606859 | 4.13E-08 |
| CTGF | ITGAM | 0.336475794 | 0.015761561 | 0.014973259 |
| FGA | ITGB1 | 0.334587275 | 0.000722062 | 0.000219547 |
| NID1 | ITGB1 | 0.331392722 | 0.002374601 | 0.000219547 |
| EDN1 | EDNRB | 0.331131381 | 0.339795861 | 6.62E-10 |
| APP | CD74 | 0.330998175 | 4.74E-09 | 0.002448836 |
| MFAP2 | NOTCH1 | 0.327072411 | 1.55E-07 | 0.392855035 |
| DLL3 | NOTCH2 | 0.326781701 | 3.89E-07 | 0.000388714 |
| BMP4 | BMPR2 | 0.325169251 | 0.618751397 | 0.00023084 |
| APP | LRP1 | 0.318987045 | 4.74E-09 | 0.048201018 |
| SEMA4C | PLXNB2 | 0.318515998 | 8.68E-06 | 0.356772193 |
| PLAT | LRP1 | 0.313722207 | 0.845235526 | 0.048201018 |
| DLK2 | NOTCH1 | 0.313118017 | 1.81E-13 | 0.392855035 |
| LTB | CD40 | 0.306854738 | 0.063284079 | 8.95E-06 |
| S100A8 | TLR4 | 0.302658591 | 3.78E-09 | 0.835793373 |
| SORBS1 | ITGB5 | 0.300690677 | 7.13E-09 | 0.331821689 |
